# Loss of Heterozygosity associated with ubiquitous environments in yeast

**DOI:** 10.1371/journal.pgen.1011692

**Published:** 2025-05-12

**Authors:** Nikilesh Vijayan, Sameer Joshi, Praseetha Sarath, Koodali T. Nishant

**Affiliations:** 1 School of Biology, Indian Institute of Science Education and Research Thiruvananthapuram, Trivandrum, Kerala, India; 2 Center for High-Performance Computing, Indian Institute of Science Education and Research Thiruvananthapuram, Trivandrum, Kerala, India; University of Strasbourg, FRANCE

## Abstract

The effect of ubiquitous environmental conditions on mutational mechanisms, particularly loss of heterozygosity (LOH) remains poorly understood. Environment induced LOH can rapidly alter the genome and promote disease progression. Using mutation accumulation (MA) lines, we analysed the effect of ubiquitous environmental conditions on mutational mechanisms in a diploid hybrid (S288c/YJM789) baker’s yeast strain. These included blue light, low glucose (calorie restriction), oxidative stress (H_2_O_2_), high temperature (37°C), ethanol, and salt (NaCl). The frequency of LOH increased significantly in all environments including calorie restriction relative to the control (YPD). Interestingly, the percentage of the genome covered by LOH varied significantly depending on the condition. For example, the LOH tracts seen in calorie restriction conditions were significantly shorter than those observed in blue light exposure that rapidly homozygotized the genome. We also report a unique mutational signature of blue light exposure comprising LOH, small indels, large deletions and transversion mutations (G:C > T:A; G:C > C:G), with the latter likely to result from the photooxidation of guanine bases. Our results suggest ubiquitous environmental conditions cause LOH but result in distinct mutational signatures due to the type of damage induced and the pathways used to repair them.

## Author summary

Loss of heterozygosity (LOH) promotes tumorigenesis by homozygotization of recessive loss of function alleles (e.g., mutated tumor suppressor alleles). The effect of ubiquitous environmental conditions on LOH is poorly understood. We show that chronic exposure to environments like blue light, calorie restriction, oxidative stress (H_2_O_2_), high temperature (37°C), ethanol, and salt (NaCl) causes enhanced LOH relative to the control (YPD). Further, the LOH signatures were also environment dependent, where blue light causes long LOH tracts while a calorie restricted environment results in short LOH tracts. As a result, blue light exposure causes extensive genome homozygotization, unlike calorie restriction. The extensive LOH caused by blue light exposure was largely due to enhanced DNA breaks generated likely through production of reactive oxygen species. Further, we discover a unique mutational signature of blue light exposure comprising enhanced LOH, small indels, large deletions and transversion mutations as a consequence of oxidative DNA damage. Our results suggest ubiquitous environments significantly affect LOH and mutation spectrum through distinct mechanisms.

## Introduction

Mutations arise from both external environmental factors and intrinsic cellular metabolic processes. Understanding how ubiquitous environmental factors cause mutations is essential, as mutations not only drive evolutionary processes but also contribute to diseases such as cancer and aging. Mutations causing extensive LOH, chromosomal rearrangements or aneuploidies create large-scale genomic changes that are also defined as genome instability. Further, LOH can homozygotize recessive deleterious alleles, including mutated tumor suppressor genes, through the loss of wild-type alleles, thereby promoting tumor development [1,2]. LOH is defined as the loss of one parental allele at a specific locus, resulting in a region of homozygosity within a diploid heterozygous genome. LOH can occur through copy number-neutral LOH, which arises from mitotic recombination events such as gene conversions or crossovers, and copy number-loss LOH, typically associated with deletions. LOH can be classified as interstitial and terminal LOH based on the nature and extent of homozygosity. LOH events occurring throughout the genome are termed interstitial LOH, whereas LOH events extending till the end of the chromosomes and involving telomeres are called terminal LOH. Interstitial LOH are predominantly induced by gene conversions or double crossovers, typically resulting in the exchange of less than 10 kilobases of chromosome segments in yeast [3,4]. Terminal LOH, is associated with reciprocal crossovers or break induced replication. This process can result in LOH tracts that span over 100 kilobases and reach the end of the chromosome in yeast [3,4]. In mammals these LOH tract lengths may extend to several megabases of DNA [5]. Furthermore, the genomic distributions of interstitial and terminal LOH events differ substantially, suggesting mechanistic differences in their initiation and resolution [6].

Recent mutation studies in the baker’s yeast *Saccharomyces cerevisiae* have shown that LOH rates far exceed point mutation rates. Interstitial LOH occurs at frequencies ranging from 0.3 to 5.6 × 10^−2^ per cell division, while terminal LOH is observed at rates of 1.4 to 9.3 × 10^−2^ per cell division [3,6,7]. When calculated on a per SNP basis, the overall LOH rate ranges between 2.6 and 7.1 × 10^−5^ per SNP per cell division, which is orders of magnitude higher than the point mutation rate of 1–3 × 10^−10^ per base pair per cell division in diploid yeast [6–10]. While the LOH rates are sensitive to the SNP density, these numbers suggest LOH as an important driver of genomic alterations, with critical implications for understanding its role in genome evolution and disease.

The genome-wide impact of environmental factors on LOH remains largely unexplored. Environmental factors such as blue light, sodium chloride (NaCl), low glucose, high temperature, and ethanol are ubiquitous for the model organism *S. cerevisiae* and have been studied in diverse contexts [11–16]. Blue light, for instance, damages DNA both directly and indirectly through reactive oxygen species (ROS) [15–18]. High NaCl levels, such as those encountered in marine ecosystems or kidney medullary cells, induce DNA breaks [14,19]. Ethanol exposure generates oxidative and replication stress, promoting mutations via error-prone DNA polymerases [13,20,21]. High temperatures shorten telomeres and alter recombination landscapes [12,21–23], while low glucose enhances DNA repair mechanisms, prevents oxidative stress and promotes longevity in simple and higher eukaryotic organisms [11,24–26].

Previous studies analysing the effect of environmental factors in *S. cerevisiae* have predominantly relied on reporter-based assays or homozygous backgrounds, limiting their ability to detect genome-wide LOH events [13,27,28]. While these studies have contributed significantly to understanding environmental mutagenesis in *S. cerevisiae*, the lack of genome-wide analyses in heterozygous backgrounds leaves a critical gap in our knowledge.

A heterozygous background reflects the genetic complexity of natural diploid populations and is essential for studying copy number-neutral LOH events. Approximately, 63% of natural diploid isolates of *S. cerevisiae* exhibit heterozygosity, highlighting its evolutionary and adaptive importance [29]. Heterozygous strains also provide a more complex genetic framework, introducing diverse allelic interactions and variation. In recent years, hybrid *S. cerevisiae* strains made by crossing S288c with strains like YJM789 or SK1 that show high divergence (0.6–0.7%) have been used to analyse spontaneous LOH through whole genome sequencing of MA lines [6,8,30–33]. However, these studies were conducted independently of environmental factors and therefore lack an understanding of how environmental factors influence LOH. In this study, we systematically examined the effects of seven environmental factors: YPD, ethanol, NaCl, temperature (37°C), low glucose, H_2_O_2_ and blue light (λ = 470 nm), on the genome-wide mutational landscape, including LOH, in the diploid hybrid strain (S288c/YJM789) of *S. cerevisiae*. Hydrogen peroxide (H₂O₂), a known source of oxidative stress that enhances LOH and point mutations [34], was included to contextualize the mutagenic potential of the other environments. Our findings reveal that blue light, considered relatively innocuous, induces extensive LOH and genomic instability in *S. cerevisiae*. Additionally, we demonstrate calorie restriction mitigates LOH tract lengths, providing insights into mechanisms that preserve genomic integrity under low glucose conditions. This work fills critical gaps in our understanding of how ubiquitous environments shape LOH patterns, with implications for both genome stability and evolutionary biology.

## Results

### Fitness assessment and propagation of *S. cerevisiae* (S288c/YJM789) MA lines under diverse environmental conditions

**Fig 1:**
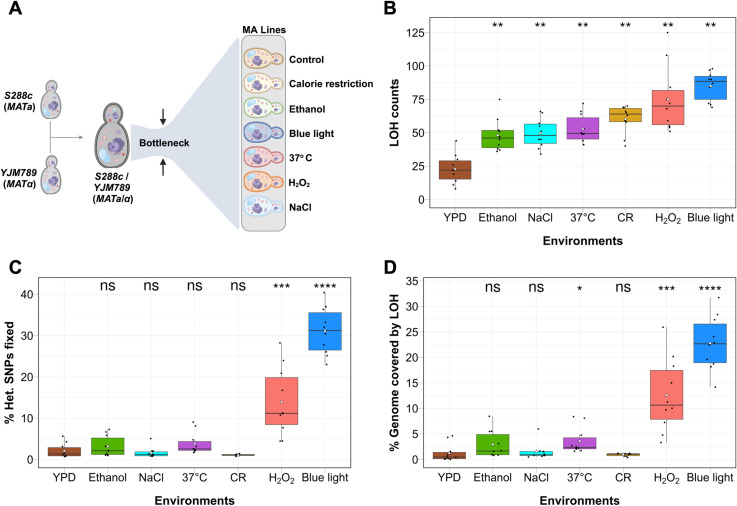
LOH profiles vary across environments. **A)** Outline of MA experiment in seven environmental conditions. 112 MA lines of the S288c/YJM789 hybrid were propagated for ~ 1000 generations. **B)** Number of LOH events across seven environmental conditions. **C)** Percentage of heterozygous SNP’s fixed under seven different environmental conditions. **D)** Percentage of the genome covered by LOH across the seven environments. **B-D)** Black dots represent the data for each line. Triangles indicate the average value. Statistical differences compared to YPD were assessed by Wilcoxon rank-sum test (* p < 0.05, ** p < 0.01, *** p < 0.001, **** p < 0.0001) followed by Bonferroni correction. ns indicates not significant. Fig 1A created in BioRender. Vijayan, N. (2025) https://BioRender.com/k46v278.

Fitness measurements of the parent *S. cerevisiae* hybrid strain (S288c/YJM789) were conducted to measure growth rate changes in seven different environments to adjust the bottleneck intervals and ensure a comparable number of generations. The seven different environmental conditions include: calorie restriction (CR, 0.05% glucose; [35,36]), 6% ethanol, 0.5 M NaCl, Blue light (λ = 470 nm, 500 μmol·m−2·s−1 photon flux density), 3 mM H_2_O_2_ and high temperature (37°C), with nutrient rich media (YPD) serving as an untreated control condition (Fig 1A). Fitness was determined using a combination of spot assays and growth rate assays (S1A and S1B Fig). Cell viability after 48 hours in all environments was comparable to the control condition (YPD) (S1A Fig). The initial growth rate was reduced for all the environments by not more than 50% relative to the control, except for high temperature (37°C) (S1B Fig). The high-temperature environment (37°C) did not affect the growth rate, presumably due to YJM789 genetic background being adapted to high temperature growth [37]. The growth rate data are shown in S1 Table.

Sixteen parallel MA lines were propagated from the parent S288c/YJM789 hybrid with exposure to the seven environmental conditions, resulting in a total of 112 MA lines. Colonies were streaked to generate single cell bottlenecks every 24, 36 or 48 hr based on the initial growth rate in each environment to minimize selection and ensure approximately 1000 generations for each environment (S1 Table). 111 MA lines remained viable at the end of the final bottlenecks as one line (CR condition) went extinct, most likely due to the accumulation of deleterious mutations. After the final bottlenecks, the growth rate was significantly altered across all environmental conditions except H_2_O_2_ (S1B Fig and S1 Table). Spore viability was analysed in five MA lines from each environmental condition after the final bottleneck. Only blue light exposure markedly reduced spore viability (mean spore viability: 17%), while all other environments maintained spore viabilities above 80%, comparable to the parent hybrid strain (90%) (S1C Fig and S2 Table). Mitochondrial function was largely preserved, as all lines except two (one each from blue light and H₂O₂ exposure) retained the ability to metabolize lactate as the sole carbon source after ~1,000 generations (S2 Fig). We sequenced 10 MA lines from each environment (total 70 lines) to investigate the genetic alterations using a short read Illumina platform at high coverage (minimum 100X). The sequence data were analysed for LOH, single nucleotide mutations (SNMs), insertions or deletions (indels), and numerical aneuploidy (Materials and Methods).

### Extensive LOH in blue light exposure

LOH tracts were identified from the short read sequence data if supported by ≥ 3 SNPs (Material and Methods, [4,32]). Approximately 52,000 heterozygous SNPs genotyped in the S288c/YJM789 parent were analysed in the MA lines under each environmental condition (S3 and S4 Tables and S3A Fig). Mean LOH counts (calculated as the average number of LOH events across 10 MA lines per environment) and LOH rates (per generation) were significantly enhanced across all environments compared to YPD suggesting environmental factors can trigger LOH (Figs 1B and S3B, Tables 1 and S5). The mean LOH rate in YPD (2.3 x 10^−2^ per generation) was consistent with estimates from previous studies [6–10]. Enhanced LOH counts across all environments relative to YPD were robust to even more conservative requirements for LOH identification (≥5 or ≥ 10 SNPs supporting a LOH event) (S3C and S3D Fig). Exposure to blue light induced maximum enhancement in LOH events across all environments tested. Blue light exposure caused an approximately four-fold increase in the mean LOH counts and LOH rate compared to the control, covering nearly 23% of the genome (calculated as the total LOH tract size of an MA line divided by the genome size) and fixing 31% of heterozygous SNPs across the genome (Figs 1B-D, S3B, Tables 1 and S4). Similarly, H₂O₂ exposure resulted in an approximately three-fold increase in the LOH events and LOH rate covering 12.5% of the genome and fixing 13.9% of heterozygous SNPs (Figs 1B-D, S3B, Tables 1 and S4). These results highlight the potential for blue light to cause significant LOH.

**Table 1:** LOH count, percentage of genome covered by LOH, LOH tract size, percentage of SNPs fixed, and the LOH rate in seven different environmental conditions.

Environment (N = 10 lines per environment)	LOH count		Genome covered by LOH (%)		LOH tract size (Kb)		Percentage SNPs fixed	LOH rate per generation
	Mean	Median	Mean	Median	Mean	Median	Mean	Mean
**YPD (Control)**	22.9	22	1.27	0.47	6.6	0.23	2.08	0.023
**Ethanol**	48.2	46	2.93	1.64	7.3	0.81	3.2	0.047
**NaCl**	49.7	48	1.51	0.90	3.6	0.72	1.6	0.048
**High temperature (37 °C)**	52.9	49.5	3.61	2.36	8.2	0.75	3.8	0.048
**CR**	60.3	64	0.93	1.02	1.85	0.5	1.07	0.06
**H**_**2**_ **O**_**2**_	75.1	70	12.5	10.6	20.1	2.5	13.9	0.069
**Blue light**	85.1	88.5	22.7	22.6	32.1	5.2	31.1	0.077

Mean LOH count is the average number of LOHs after ~1000 generations for the 10 MA lines/environment.

In contrast to blue light and H₂O₂, CR condition increased the mean LOH counts and LOH rate by approximately three-fold but the percentage of the genome covered by LOH (0.93%) and heterozygous SNPs fixed (1.07%) were similar to that of the control (Fig 1B-D, S3B, Tables 1 and S4). Other environmental conditions: ethanol, NaCl, and high temperature showed an approximately two-fold increase in the mean LOH counts and LOH rate with modest enhancement in the percentage of genome covered by LOH (1.5% to 3.6%) relative to the YPD control (1.27%) (Fig 1B, 1D and Table 1). The percentage of heterozygous SNPs fixed in these three environments (1.6% to 3.8%) was also similar to the YPD control (2.08%) (Fig 1C, Tables 1 and S4). These results suggest limited capacity of CR, ethanol, NaCl, and high temperature for driving large-scale genomic homozygosity compared to blue light and H₂O₂ (Fig 1B-D, Tables 1 and S4).

We also observed from the pooled LOH map across all environments that the frequency of LOH events rises with increasing distance from the centromere (S4A Fig). Centromere proximal LOH arises mostly through gene conversion generating interstitial LOH events, and centromere distal events through crossover and break induced replication pathways generating terminal LOH tracts [6,31]. The average distance of LOH tracts from the telomere was much shorter than the distance from the centromere across all environments (S5 Fig and S6 Table). These results may reflect enhanced mitotic recombination rates in centromere distal regions. Alternatively, mitotic recombination rates may be the same everywhere on the chromosomes, but telomere proximal SNPs are more susceptible to LOH from recombination events initiating far away.

Although our results show enhanced LOH in the environments analysed in this study, it is important to note that the LOH estimates are conservative across all environments. This is because, although heterozygous SNPs cover the entire chromosome, the SNP density along the chromosome varies. The average SNP density is 6.1 per kb [37] but some chromosomal regions have higher and lower SNP densities (S4A Fig). We analysed LOH counts and SNP density distribution along the chromosomes (S4A Fig). With a requirement of 3 SNPs to call a LOH tract, we are likely to miss short LOH tracts in regions with low SNP density. At the same time regions with extremely high SNP density (e.g., Chr I- 180–190kb) also showed less LOH, possibly due to rejection of strand invasion intermediates (S4A Fig).

### LOH tracts are long in blue light and H_2_O_2_ exposure and short in CR environment

**Fig 2:**
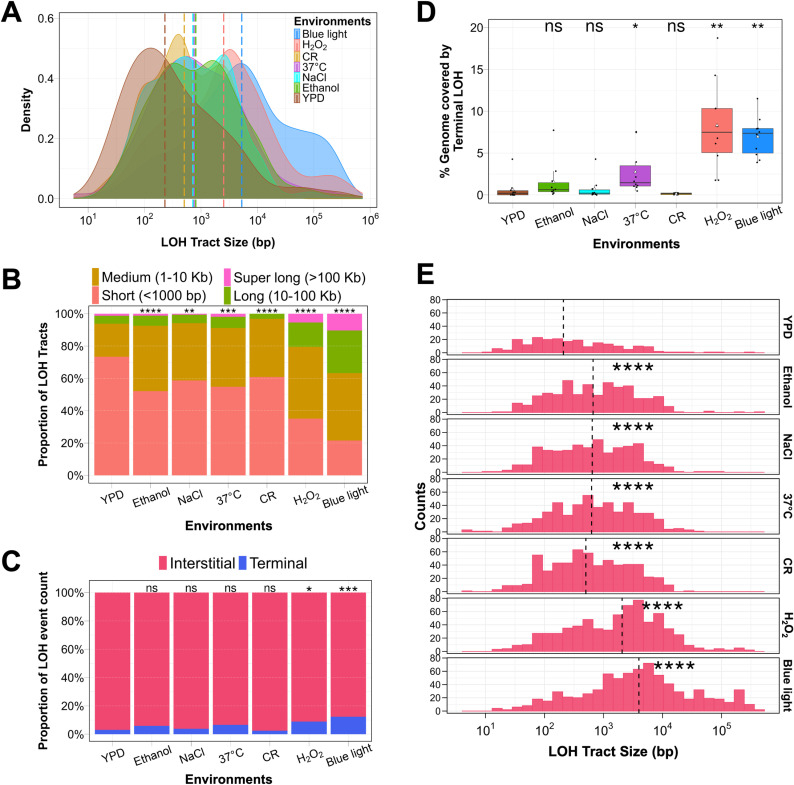
Environmental modulation of LOH tract size and distribution. **A)** Density plot showing the distribution of LOH tract size for all the environments. Vertical dotted lines show the median LOH tract size for each environment. LOH tract sizes in each environment significantly differed from the YPD control (p < 0.0001). LOH tract size for CR were significantly shorter than blue light and H_2_O_2_ (p < 0.0001). Statistical significance was assessed by the Wilcoxon rank-sum test followed by Bonferroni correction. **B)** Stacked bar plot shows the proportion of LOH counts based on tract size in percentage for: short tracts (< 1000 bp), medium tracts (1000 – 10,000 bp), long tracts (10,000 – 100,000 bp), and super long tracts (> 100,000 bp). **C)** Proportion of interstitial and terminal LOH events across seven different environments. For panels **B** and **C**, Chi-Square test with Bonferroni correction was used to assess statistical significance relative to YPD control (* p < 0.05, ** p < 0.01, *** p < 0.001, ****p < 0.0001). ns indicates not significant. **D)** Percentage of the genome covered by terminal LOH events across seven different environments. Black dots show the data for each MA line. Triangles show the average value in each environment. **E)** Histogram represents the distribution of interstitial LOH events in each environment based on tract size. Vertical dotted lines represent the median interstitial LOH tract size. For panels **D** and **E**, statistical significance was assessed using the Wilcoxon rank-sum test with Bonferroni correction (* p < 0.05, ** p < 0.01, **** p < 0.0001). ns indicates not significant.

Although exposure to blue light, H_2_O_2_ and CR environments enhanced mean LOH counts and rates three to four fold, their effects on the percentage of the genome fixed by LOH were markedly different (Fig 1B-D and Table 1). These observations encouraged us to analyse the LOH tract sizes in the seven environmental conditions (Fig 2A and Table 1). Compared to the YPD control, the density plot for LOH tract size distribution was skewed to the right for all environmental conditions (Figs 2A, S6A and S6B). The median and mean LOH tract sizes for CR condition was significantly shorter than H_2_O_2_ and blue light exposure (Table 1, Figs 2A, S6A and S6B). These results suggest that while blue light and H_2_O_2_ exposure result in long LOH tracts, LOH events in calorie restricted condition are biased toward short tracts. These results also explain why the percentage of SNPs fixed and genome covered by LOH in CR is comparable to the control, even though LOH counts in CR are three fold higher than control.

LOH tracts were further categorized based on size as short tracts (less than 1 kb), medium tracts (ranging from 1 kb to 10 kb), long tracts (ranging from 10 kb to 100 kb), and super-long tracts (more than 100 kb) (Figs 2B and S6C). Since LOH counts were highly variable across the environments, we compared the proportion of LOH events in each size category instead of absolute numbers. Blue light exposure showed a significant increase in long (10–100 kb) and super-long (> 100 kb) LOH tracts, which were five to seven-fold higher than in control, promoting rapid homozygotization of the genome (Figs 2B, S6C and S6 Table). H₂O₂ exposure showed a similar pattern, with a preference for long and super-long tracts, though to a lesser extent than blue light (Figs 2B, S6C and S6 Table). In contrast, LOH tracts in the calorie-restricted condition showed a distinct bias toward short-tract LOH (< 1 kb) (S6C Fig and S6 Table), which occurred at nearly twice the proportion of that in blue light and H₂O₂ exposure (Figs 2B, S6C and S6 Table). This shift resulted in a reduced proportion of long-tract LOH and an absence of super-long LOH tracts in CR condition (Figs 2B, S6C and S6 Table). This strong preference for short-tract LOH likely helps limit extensive genome-wide homozygotization in calorie-restricted conditions. Other environments, such as ethanol, NaCl, and high temperature, also showed a significant increase in LOH counts (two fold higher) but did not show the same preference for long/ super long LOH tracts as observed in blue light or H₂O₂ (Figs 1B, 2A, 2B, S6 and Table 1). Consequently, the percentage of the genome covered by LOH in these conditions showed only modest increases compared to the control, with the increase in NaCl and ethanol being statistically insignificant (Fig 1D and Table 1).

**Fig 3:**
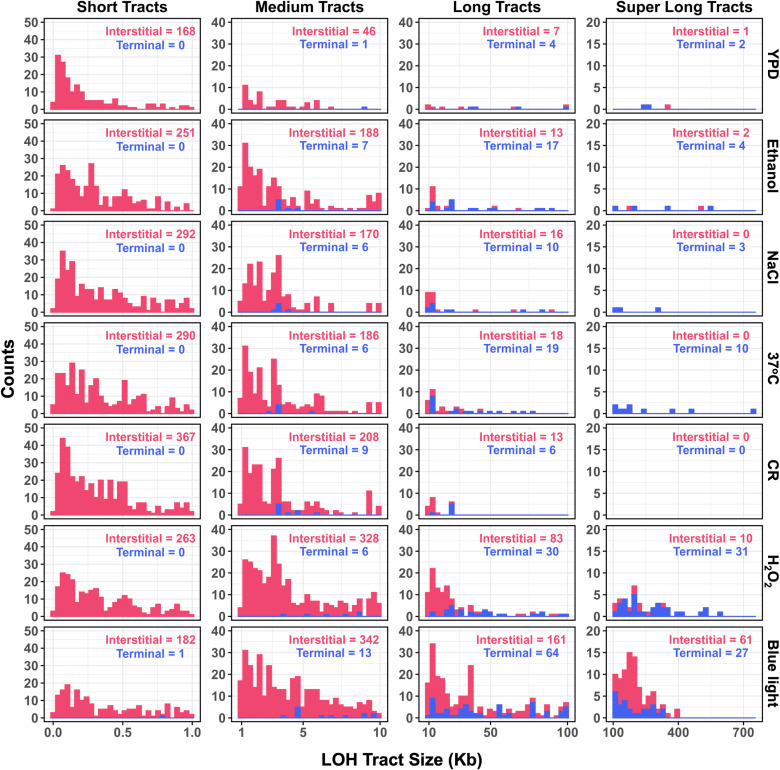
Distribution of interstitial and terminal LOH tracts based on LOH tract length. Histogram represents the distribution of interstitial and terminal LOH counts across different categories of LOH tract sizes. LOH counts are shown for short tracts (< 1000 bp), medium tracts (1000 – 10,000 bp), long tracts (10,000 – 100,000 bp), and super long tracts (> 100,000 bp).

To understand the mechanisms driving the differences in LOH tract sizes across environmental conditions, we analysed the distribution of interstitial and terminal LOH tracts. The distribution of interstitial and terminal LOH events in the S288c/YJM789 hybrid used in this study was similar to other studies with interstitial LOH being more centromere proximal and uniformly distributed compared to the terminal LOH events (S4B and S4C Fig) [6,7,38]. Consistent with prior studies that show mitotic crossovers are generally suppressed [6,7,38,39], we observed that interstitial LOH events outnumbered terminal LOH events in all environments (Fig 2C and S6 Table). Further, interstitial LOH tract sizes significantly increased across all environments relative to YPD (median size, 0.2 kb) with blue light exposure showing the longest interstitial LOH tract size (median size, 3.9 kb) (Fig 2E and S6 Table). We also observed that the proportion of terminal LOH events was elevated in blue light and H_2_O_2_ exposure, compared to YPD (Fig 2C, 2D and S6 Table). The elevated proportion of terminal LOH tracts in blue light exposure was not simply because of the abundance of long (10–100 kb) and super-long (> 100 kb) LOH tracts (Figs 2B and S6C). Analysis of the distribution of interstitial and terminal LOH tracts based on LOH tract length showed that many super long LOH tracts in blue light exposure were interstitial than terminal (Fig 3). These results showing long interstitial LOH tracts (Figs 2E and 3) and enhanced proportion of terminal LOH tracts (Fig 2C and 2D) in blue light exposure suggest differences in the processing of mitotic double strand breaks (DSBs) compared to the YPD control (see discussion). These findings underscore the varying genomic effects of different environmental conditions, where blue light and H₂O₂ promote genome homozygotization through long LOH tracts, while CR mitigates it by favouring shorter LOH tracts.

### SNM rate and spectrum are altered in blue light exposure

**Fig 4:**
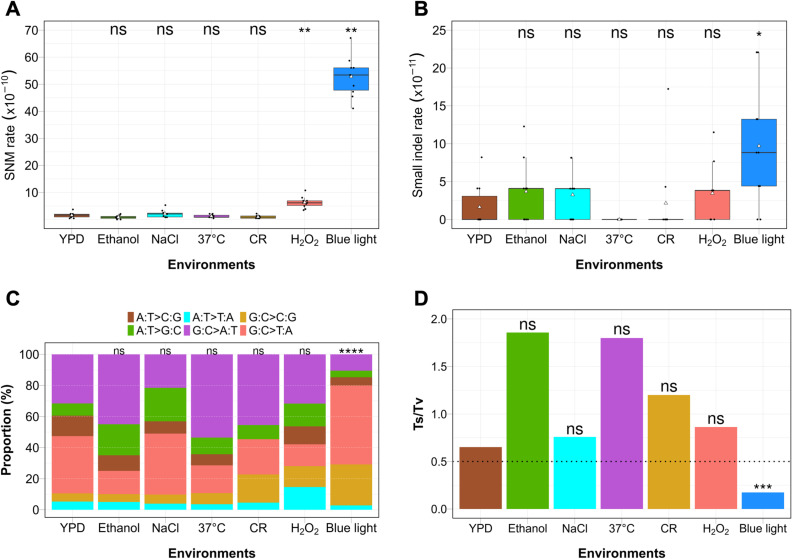
Mutation rate and spectrum vary across environments. **A)** SNM rates and **(B)** small indel rates for each environment were measured by dividing the total number of mutations per line by the genome size and number of generations. Black dots show the SNM/indel rates in individual MA lines. Triangles indicate the average SNM/indel rate in each environment. **C)** Proportion of all six type of SNMs in each environment including transition (A:T > G:C; G:C > A:T) and transversion (A:T > C:G; A:T > T:A; G:C > C:G; G:C > T:A) mutations. **D)** Ratio of transition (Ts) to transversion (Tv) mutations in each environment. In the absence of a mutational bias, a Ts/Tv ratio of 0.5 is expected (horizontal black dotted line). Panels **A** and **B** were analysed for statistical significance using Wilcoxon rank-sum test with Bonferroni correction (* p < 0.05, ** p < 0.01). ns indicates not significant. Panels **C** and **D** were analysed for statistical significance using Chi-Square test with Bonferroni correction (*** p < 0.001, **** p < 0.0001). ns indicates not significant.

We analysed SNMs and small indels across the seven environments. The SNM (1.56 x 10^−10^) and small indel (0.16 x 10^−10^) rates per bp/generation for the S288c/YJM789 hybrid in YPD were similar to the SNM (1.95 x 10^−10^, S288c), (0.98 × 10^−10^, S288c/YJM789) and small indel (0.19 x 10^−10^, S288c) rates per bp/generation previously reported in the literature for the *S. cerevisiae* strains in YPD [27,30] (Figs 4A, 4B, SA7 and S7B Table). Blue light exposure significantly increased SNM rates by 34 fold (52.8 x 10^−10^/bp/generation) and small indel rate by six fold (0.97 x 10^−10^/ bp/ generation) compared to YPD (Fig 4A, 4B, S7A and S7B Table). This increase was likely driven by photo-oxidation of guanine bases, resulting in a significantly distinctive mutational signature characterized by transversions, specifically G:C > T:A and G:C > C:G mutations (Fig 4C and S7C Table). In the absence of a mutational bias a Ts/Tv ratio of 0.5 is expected since there are two types of transition (A:T > G:C; G:C > A:T and four types of transversion (A:T > C:G; A:T > T:A; G:C > C:G; G:C > T:A) mutations. The Ts/Tv ratio for blue light was markedly reduced to 0.17, indicating an excess of transversions, unlike the control and other environments where transitions were more frequent (i.e., Ts/Tv ratio> 0.5) (Fig 4D and S7C Table). Similarly, H₂O₂ exposure showed a four-fold increase in SNM rate (6.29 × 10^−10^/bp/generation) (Fig 4A and S7A Table). However, the indel rate in H₂O₂ (0.34 x 10^−10^) was comparable to the control (Fig 4B and S7B Table). For all other environmental conditions (CR, high temperature, ethanol and NaCl exposure), the SNM and small indel rates were comparable to the YPD control (Fig 4A, 4B, S7A and S7B Table). A subset of seven SNMs and three small indels were verified using Sanger sequencing (S7 Fig). We also tested for any bias towards GC versus AT mutations. Usually, there is a bias towards AT mutations (AT/GC > 1) in all species examined [40,41]. A similar bias was observed in exposure to all seven environments (S7C Table).

### Aneuploidies are comparable across all environments

Numerical aneuploidy involves either the gain or loss of chromosomes. Aneuploidy was detected from read coverage and verified using allele frequency data (See methods). In the control condition, only one MA line showed numerical aneuploidy for chromosome 9 (gain of chromosome) (S8B Fig). No aneuploidy was observed in high temperature environment (S8E Fig). A similar low frequency of aneuploidy was observed in blue light exposure (gain of chromosomes 9 and 14), H_2_O_2_ (loss of chromosome 1), ethanol (gain of chromosome 3), and CR (gain of chromosome 4) (S8C, S8F, S8G and S8H Fig). Aneuploidy was enhanced six fold for NaCl exposure (S8A, S8D Fig and S8 Table) but not statistically significant consistent with previous studies [27]. 13 numerical aneuploidy events, mostly involving small chromosomes were observed, with 11 involving the gain of chromosomes (chromosomes 1, 3, 4, 6, 9, 14) and two being the loss of chromosomes (chromosomes 1, 3) (S8 Table). Overall, considering all the environmental conditions, including control, it was observed that chromosomes 1, 3, 6 often underwent aneuploidy. These results are consistent with previous studies [29,42], which showed frequent aneuploidy of chromosomes 1, 3, and 6. It was also observed previously that aneuploidy events have a significant negative correlation with chromosome size [29,42].

### Structural variations involving large deletions are enhanced in blue light exposure

**Fig 5:**
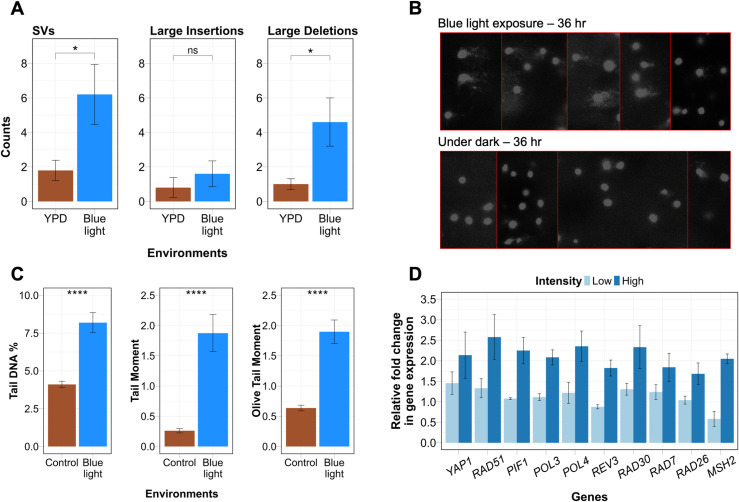
Chronic blue light exposure affects DNA integrity and expression of DNA repair genes. **A)** Structural variant events comprising large deletions and insertions > 50 bp in control and blue light exposure. Data represent mean ± SE. **B)** Acellular alkaline comet assay on lysed yeast cells irradiated with blue light (470 nm, 500 μmol·m−2·s−1) for 36 hr. Representative images show nuclei stained with propidium iodide following blue light and dark exposure for 36 hrs. **C)** The Tail DNA percentage, Tail Moment and Olive Tail Moment parameters used to assess the extent of direct DNA damage. Data represent mean ± SE obtained from three independent biological replicates. **D)** Gene expression profile of candidate genes following blue light exposure for 36 hrs at low (500 μmol·m−2·s−1) and high (920 μmol·m−2·s−1) intensity. The bar graph shows fold change relative to *GAPDH* calculated using 2^-∆∆Ct^ method. The fold change and standard error are shown from three independent biological replicates. Panels **A** and **C** were analysed for statistical significance using the Wilcoxon rank-sum test (* p < 0.05, **** p < 0.0001). ns indicates not significant.

Since the S288c/YJM789 hybrid exposed to blue light showed extensive genetic changes, we performed long read sequencing to detect structural variations that may be hard to observe from short read sequence data. Five lines from the YPD control and blue light exposure were sequenced using Oxford nanopore sequencing to quantify structural variations greater than 50 bp in size. 23 large deletions were observed under blue light exposure, compared to five in the control condition, primarily within the size range of 50–1000 base pairs (Fig 5A and S9 Table). Insertions, however, were comparable between blue light (8) and control (4) conditions, indicating that large deletions are the predominant structural variation under blue light exposure (Fig 5A and S9 Table). Some of the large deletions were also confirmed using the short read Illumina sequence data from the absence of short reads mapping to the deleted regions.

Since large and small deletion events were enhanced, especially in blue light exposure (Figs 4B and 5A), we analysed LOH events based on probable mechanism (deletion or mitotic recombination) (Materials and Methods) in all environments. The proportion of LOH events from recombination was similar in YPD (69%), ethanol (69%), high temperature (71%) and NaCl exposure (73%) (S5 Table and S9 Fig). The proportion of LOH events from recombination was elevated in H_2_O_2_ (82%) and blue light (91%) exposure compared to YPD (69%), while it was slightly reduced in calorie restriction (61%) (S5 Table and S9 Fig). Overall, most LOH events were likely to arise from recombination rather than deletion consistent with previous studies [6,32].

### DNA breaks are enhanced in blue light exposure

Among all the seven environments, exposure of cells to blue light generated extensive LOH, which may be due to the generation of DNA breaks. To quantify the extent of DNA damage induced by blue light on yeast cells, an acellular alkaline comet assay was performed. It is a modified version of the standard alkaline comet assay in which cells embedded in agarose were lysed before exposure. The acellular nature of the assay measures damage specifically to the naked DNA in a supercoiled structure, eliminating the occurrence of DNA repair. The DNA-embedded slides were irradiated with blue light for 36 hr, while the control slides were kept in the dark. We selected a 36 hr exposure window, consistent with the bottleneck interval for MA lines propagated under blue light exposure. The extent of the DNA damage (i.e., the fragmented DNA) is reflected in the length and the intensity of the tail part of the comet. We used Tail DNA percentage (percentage of the total DNA present in the tail), Tail Moment (Tail length × %Tail DNA) and Olive Tail Moment (distance between the centre of the head and tail of the comet x percentage of Tail DNA) parameters to assess the damage induced by blue light irradiation (see materials and methods). All these parameters were significantly enhanced in blue light-exposed samples (Fig 5B and 5C), which implies that blue light causes strand breaks that result in enhanced LOH. Previous studies have also shown a direct impact of blue light on the integrity of genomic DNA in mouse cell lines during long term exposure [16].

### Chronic blue light exposure modulates expression of oxidative stress response and DNA repair genes in a dose dependent manner

Although, *S. cerevisiae* lacks photoreceptors, it senses blue light by converting the light energy into H_2_O_2_ with the help of peroxisomal oxidase [43]. To investigate the molecular basis of blue light-induced genomic instability, we analysed the expression of genes involved in oxidative stress response and DNA repair. These genes were selected based on their established roles in responding to oxidative stress (e.g., *YAP1*), repairing DSBs (e.g., *RAD51*, *POL3*, *PIF1*), bypassing DNA lesions (e.g., *RAD30*, *REV3*), and recognizing and correcting mismatches or bulky lesions (e.g., *MSH2*, *RAD26*, *RAD7*). This targeted approach allowed us to link gene expression changes directly to the observed mutation spectrum under blue light exposure. Our analysis revealed a significant and dose dependent upregulation of oxidative stress and DNA repair pathways in response to blue light exposure. The oxidative stress regulator *YAP1* was upregulated by 1.5-fold under low-intensity blue light (500 μmol·m^−^²·s^−^¹) and by two-fold under high-intensity blue light (920 μmol·m^−2^·s^−1^) (Fig 5D and S10 Table). This result indicates that YAP1 responds proportionally to the levels of reactive oxygen species (ROS) generated during blue light exposure, consistent with its established role in oxidative stress adaptation [44].

In addition to oxidative stress response, DNA repair genes also showed significant upregulation under blue light exposure. Notably, *RAD51*, a gene involved in homologous recombination, is up-regulated by 2.6 fold under high-intensity blue light (Fig 5D and S10 Table) indicating the presence of DSBs that may result in LOH (Fig 5B). Break Induced Replication (BIR) is often used when extensive DSBs cannot be resolved through homologous recombination alone, leading to large-scale genomic changes. BIR genes such as *PIF1* and *POL3* [45,46], were up-regulated by 2.2 and 2.1 fold at high blue light intensity, suggesting that the long terminal LOH tracts observed under blue light exposure may arise through this repair mechanism (Fig 2C and 2D).

Error-prone DNA repair mechanisms were also activated, as evidenced by the upregulation of *REV3* (1.8-fold) and *RAD30* (2.3-fold) [13,27,47], encoding translesion synthesis polymerases that enable replication across damaged templates (Fig 5D and S10 Table). These findings correlate with the elevated SNM rates observed under blue light (Fig 4A). Additionally, the mismatch repair gene *MSH2* [48,49] was upregulated two-fold, suggesting increased repair of mismatches generated during replication or damage bypass (Fig 5D and S10 Table). Nucleotide excision repair (NER) pathways, responsible for repairing bulky lesions, showed activation under high-intensity blue light exposure. *RAD7*, associated with global genome repair, and *RAD26*, involved in transcription-coupled repair [50,51], were upregulated by 1.8- and 1.7-fold, respectively (Fig 5D and S10 Table). This highlights the role of NER in mitigating damage caused by ROS-induced lesions.

Lastly, the non-homologous end joining (NHEJ) pathway, typically involved in the repair of DSBs, also showed increased activity. The *POL4* gene [52], which is responsible for gap-filling during NHEJ, is up-regulated 2.3 fold at high blue light intensity (Fig 5D and S10 Table). This correlates with the higher frequency of small indels observed in blue light-exposed yeast (Fig 4B). Together, these findings suggest that chronic blue light exposure activates a complex network of oxidative stress response, DNA repair and damage tolerance pathways to prevent chromosomal instability.

## Discussion

### Environment and its impact on the LOH spectrum

**Fig 6:**
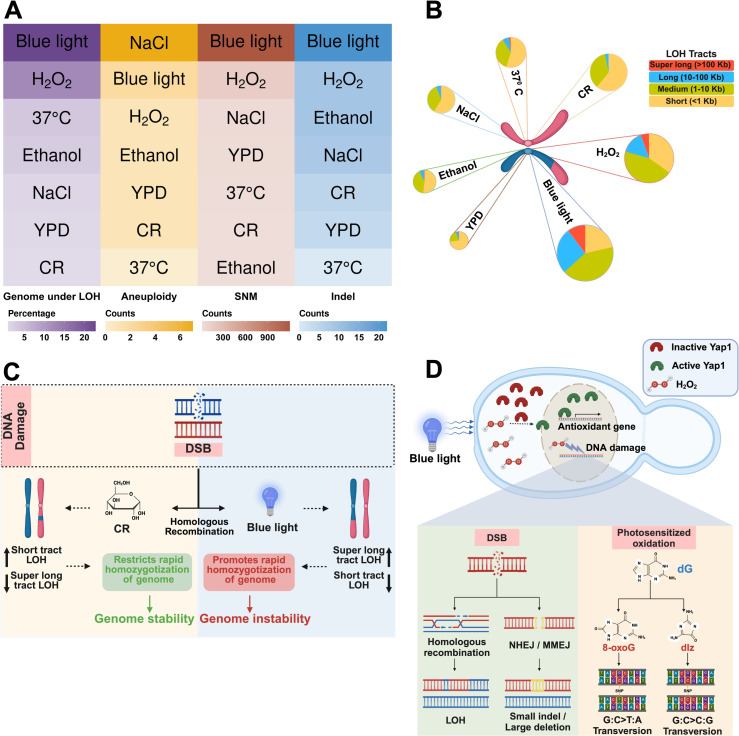
Effect of ubiquitous environmental conditions on genome stability. **A)** Mutational spectrum across seven environmental conditions. Heat map shows the quantification for each type of mutation. **B)** Environmental impact on LOH events and size. LOH events are significantly enhanced in all environments compared to the control. The proportion of LOH events based on tract size is also significantly altered across environments. **C)** Model depicting the effect of CR and blue light exposure on genome stability. Under blue light exposure, repair of DSBs by crossovers/ break induced replication causes long LOH tracts, genome homozygosity and instability. Under CR conditions, the SDSA pathway is preferred for DSB repair, favouring short LOH tracts and enhanced genome stability. **D)** Schematic representation of blue light associated mutational signature: Blue light induces oxidative stress in yeast by generating H_2_O_2_. Under blue light exposure, Yap1 is activated by H_2_O_2_, initiating oxidative-stress response. The unrepaired oxidative products of guanine, 8-oxoG and dIz form base pairs with adenine and guanine respectively resulting in enhanced transversions (G:C > T:A and G:C > C:G). Additionally, blue light induced H_2_O_2_ may cause DNA breaks primarily repaired by homologous recombination leading to enhanced LOH. Some breaks repaired through NHEJ/ MMEJ, give rise to small indels and large deletion events. Fig 6B created in BioRender. Vijayan, N. (2025) https://BioRender.com/q96z115. Fig 6C created in BioRender. Vijayan, N. (2025) https://BioRender.com/c52k078. Fig 6D created in BioRender. Vijayan, N. (2025) https://BioRender.com/c51g173.

Previous studies of environment associated mutagenesis in yeast overlooked LOH events due to the use of a homozygous background [13,27,28]. In this study we use a heterozygous background to show that ubiquitous environmental conditions can enhance LOH among other types of mutations. Blue light exposure stands out among the environmental conditions analysed in this study due to its pronounced effects on LOH (Fig 6A and 6B). The effect of blue light on cellular function depends on various factors such as wavelength, exposure intensity and duration. The primary source of the natural blue light spectrum is sunlight [53]. In the case of artificial light, the primary source are LEDs, which mostly combine light in the blue spectrum and yellow phosphor to emit light at longer wavelengths, creating the appearance of white light [54,55]. At longer wavelengths (>500 nm), the amount of energy emitted decreases, causing less oxidative stress [56]. Shorter wavelengths like UV (100–400 nm) cause DNA lesions through ROS or unrepaired thymidine dimers leading to LOH and enhanced base mutations [57–59]. Similarly blue light may also cause DNA breaks via ROS or direct damage via thymine dimers [17]. The direct impact of blue light on DNA integrity was analysed in previously reported studies where DNA breaks were observed [16,17,60]. These breaks induced by blue light can potentially induce genetic recombination and LOH in cells, which has remained unexplored until now.

Our findings reveal that blue light exposure significantly increases LOH, primarily driven by mitotic recombination events (Figs 1B, S9). Terminal LOH tracts arising from crossovers/break induced replication events were elevated in blue light exposure (Fig 2C and 2D), with tract size extending above 100 kb in length. These signatures of super long LOH tracts were significantly enhanced in blue light and H_2_O_2_ exposure compared to other environments (Figs 2A, 2B and 3). It is unlikely that the long and super long LOH tracts observed in blue light exposure reflect the merging of short tracts over the ~ 1000 generations as analysis of intermediate bottlenecks from previous studies suggests LOH events are sporadic [32]. Interestingly even interstitial LOH tracts arising primarily from gene conversions were longer in blue light exposure compared to other environments (Figs 2E and 3). This may reflect longer DSB resection or extensive branch migration followed by non-crossover resolution of the double HJs during repair. Alternatively long interstitial LOH tracts may also reflect enhanced mitotic double crossovers. Together, these observations suggest mitotic DSBs formed during blue light exposure may be repaired with a bias for crossovers than gene conversions. These results align with studies linking blue light to oxidative stress and provide mechanistic insights into studies linking blue light exposure to genomic instability and cancer (Fig 6C) [15–17,61].

In contrast, CR induced higher LOH counts but favored short LOH tracts, limiting genome-wide homozygotization (Figs 1B-D, 2A, 2B, 3 and S6C). This conservative repair mechanism likely involves synthesis-dependent strand annealing (SDSA). It may be modulated by Sir2-mediated genome silencing at telomeres, which represses mutagenic pathways like BIR and mitotic crossovers [62,63], possibly limiting extensive LOH and rapid homozygotization of the genome (Fig 6C). Moreover, although surprising, the elevated LOH events in CR also aligns with a previous study which reported ~15% increase in DSBs in wild *S. cerevisiae* strains on medium containing 0.05% glucose (CR) compared to medium containing 2% glucose [26]. These observations reinforce CR’s role as a stressor that enhances repair activity through conservative pathways and preserving genomic stability, consistent with its known benefits in promoting longevity and reducing oxidative damage [23,25,63,64].

High temperature (37°C) was observed to enhance LOH events and increase the percentage of the genome made homozygous by LOH. Notably, temperature stress promoted the formation of terminal LOH tracts (Fig 2D). This enhancement in terminal LOH may result from telomere shortening in yeast, a phenomenon known to occur at physiological body temperature (37°C) [21]. Shortened telomeres can elicit DNA damage response and activate homologous recombination mediated by Rad51-dependent and Rad51-independent pathways [65,66]. These pathways may facilitate the generation of terminal LOH tracts through BIR and mitotic crossover events [58,67]. Previous studies have shown that even a short duration of heat shock (52°C) can stimulate mitotic recombination in *S. cerevisiae* [23]. While yeast cells in the wild or during fermentation processes or infection do not experience such high temperatures, these studies may explain enhanced LOH events at 37°C (Fig 1B).

Both ethanol and salt stress increased LOH counts, however the percentage of the genome covered by LOH remained comparable to the control (Fig 1D). The effect of ethanol was likely mediated through its metabolism to acetaldehyde- a known carcinogen associated with formation of DNA adducts, DNA-interstrand crosslinks, DNA-protein crosslinks and, DSBs that elevate cancer risk [13,68]. Previous studies have reported an increase in sister chromatid recombination following exposure to ethanol and acetaldehyde [68,69]. However, these studies did not examine inter -homolog recombination events that can lead to LOH. Our results suggest that the enhanced LOH observed under ethanol exposure may also contribute to carcinogenesis in other organisms. Enhanced LOH under salt stress was likely due to NaCl-induced DSBs [14,70]. Notably, an earlier study on the mutagenic effects of NaCl did not report LOH as it used homozygous strains [27]. These findings also have evolutionary implications as NaCl-induced LOH could shape genetic diversity and adaptation in heterozygous organisms exposed to high-salinity environments. Our results suggest ubiquitous environmental conditions enhance LOH events and affect the choice of repair pathways resulting in distinct LOH signatures in *S. cerevisiae*.

### Diverse genomic alterations under ubiquitous environments: Base mutations, indels, and chromosomal instability

Although all the environmental conditions analysed influenced LOH spectrum, some of them also enhanced point mutations, indels, and aneuploidy. Among them, blue light exposure significantly increased the base mutation rate with a unique bias towards transversions (G:C > T:A and G:C > C:G). The transversion mutations under blue light exposure likely arise from the photo-sensitized oxidization of guanine bases to 8-oxo-7,8-dihydro-guanine (8-oxoG) and 2-amino-5-[(2-deoxy-β-D-erythro-pentofuranosyl) amino]-4H-imidazol-4-one (dIz), that base excision repair fails to repair accurately (Fig 6D). Such unrepaired oxidized guanine bases can result in G:C > T:A and G:C > C:G transversions [71]. In contrast, H₂O₂ exposure also elevated base mutation rates but lacked a transversion bias, suggesting more efficient repair of oxidized bases. A previous study using a locus-specific mutation reporter demonstrated that ethanol exposure enhances base mutation rates, due to stalled replication forks that recruit error-prone polymerases [13]. While our genome-wide analysis did not observe an overall increase in base mutation rates under ethanol exposure, we observed a bias toward transitions over transversions, consistent with the findings of the previous study (Fig 4C and 4D).

Indel mutations, especially large deletions were elevated only in blue light exposure (Fig 4B). It is possible some proportion of DNA breaks in yeast cells exposed to blue light may also be repaired through the NHEJ or microhomology mediated end joining (MMEJ) pathways, giving rise to indels and large deletion events (Fig 6D). Unlike other mutation types, aneuploidy frequencies remain comparable to the control in most stress conditions, including blue light. These observations suggest that although blue light can induce spontaneous DNA breaks (Fig 5B and 5C), they are efficiently repaired by homologous recombination and prevent chromosomal instability. However, enhanced whole chromosomal aneuploidies (though statistically insignificant) was observed in salt stress (S8A Fig) similar to previous studies [27]. This is likely because high NaCl decreases homologous recombination activity by preventing the localisation of Mre11 to the DNA damage site and the phosphorylation of H2AX histone [70]. The presence of unrepaired DNA breaks due to inhibition of the homologous recombination pathway may enhance chromosome mis-segregation causing aneuploidy.

### Mechanistic insights into blue light induced genomic instability

Blue light exposure is assumed to have mild effects primarily related to the disruption of the sleep cycle and circadian rhythm [72]. This study positions blue light as a novel environmental mutagen enhancing LOH, SNMs, small indels and structural variations (Fig 6D). Blue light induced DNA breaks (Fig 5B), predominantly activates HR, as evidenced by the upregulation of *RAD51* and other HR-associated genes (Fig 5D). HR prevents chromosomal instability by repairing DSBs but also contributes to LOH through mitotic recombination. Some of the mutation signatures of blue light exposure (e.g., enhanced LOH and SNMs) were similar to those observed in MA lines exposed to H_2_O_2_ – a common metabolic product of ROS [34,73,74]. Flavin containing oxidases in peroxisomes and mitochondria are known to produce H_2_O_2_ under blue light exposure in mammalian cells [75]. Consistent with this, previous studies in yeast show that blue light exposure results in H_2_O_2_ production through peroxisomal oxidase [43] and H_2_O_2_ can activate Yap1 mediated oxidative stress response [44]. The modest upregulation of *YAP1* (~1.5 fold) (Fig 5D) under low intensity blue light exposure (500 μmol·m−2·s−1) confirms blue light as an oxidative stressor. Therefore, it is not surprising that some of the mutational spectrum of blue light exposure resembles that of H_2_O_2_. In addition to oxidative stress response and HR, blue light exposure also triggers the activation of various repair pathways. Genes involved in NHEJ, mismatch repair, and error-prone DNA synthesis pathways were upregulated, reflecting a multifaceted cellular response to blue light-induced damage. The dual activation of conservative (HR) and mutagenic (NHEJ/MMEJ) pathways that result in LOH, indels and large deletions (Fig 6D) highlights the complexity of the stress response to blue light.

In conclusion, our studies highlight an unexpected role for blue light in genomic instability in *S. cerevisiae*. In this context, it may be noted that *S. cerevisiae* is not known to have photoreceptors or utilize light to produce energy or regulate biological processes [76]. Hence *S. cerevisiae* essentially has a stress response to light. This stress response results in extensive LOH, base mutations, small indels and large deletions, which besides causing genomic instability may also accelerate evolutionary processes in *S. cerevisiae*. Our studies provide a mechanistic basis for the potential use of blue light as an anti-fungal agent, which is a safer alternative to UV and other chemicals currently used to treat fungal contamination [77]. It may be noted that LOH is also associated with increased resistance to fungicides and increased virulence in fungal pathogens [78–81]. Further, we report a novel observation that CR condition also enhances LOH events. However, the genome protective effects of CR are likely mediated through enhanced use of SDSA type mechanisms that limit LOH tract sizes preventing rapid genome homozygotization. Environmental conditions therefore trigger distinct LOH patterns and mutational signatures with consequences for genomic stability and evolution.

## Methods

### Strains and media

An S288c/YJM789 hybrid strain (KTY84 x KTY83) (*MATa ho:lys5*/ *MATα* ho::*hisG* lys2 *cyh*) was used for the MA experiments. In addition to 59,361 documented SNPs, other major differences between the S288c and YJM789 genomes include a few genes that are found in YJM789 but not in S288c; an inversion (32.5 kb) on chromosome XIV in S288c, an ~ 18 kb translocation between chromosomes VI of S288c and X of YJM789; and ~ 6000 in-del polymorphisms between S288c and YJM789 [37]. For the MA experiments, the S288c/YJM789 hybrid strain was propagated on YPD agar plates (Yeast extract-1%, Peptone-2%, Glucose-2%). Different environmental conditions included YPD agar plates with either 0.5 M NaCl, 6% ethanol, 3 mM hydrogen peroxide or with continuous exposure to blue light (λ=470 nm) (WILLS 165W LED aquarium light) at low (500 μmol·m−2·s−1) or high (920 μmol·m−2·s−1) intensity. The photon flux density of the LED aquarium light (λ = 470 nm) was measured by a photosynthetic photon flux density sensor (#HPL200P, HOPOCOLOR, Zhejiang, China). Based on the literature, YPD agar plates with low glucose (0.05%) were used for the calorie-restricted condition [36]. All lines were propagated at 30°C, except for the high temperature condition (37°C).

### Optimization of environmental conditions

A spot assay was performed to evaluate the growth and survival of the *S. cerevisiae* hybrid (S288c/YJM789) under various environmental conditions [82]. For the spot assay, yeast cells were streaked in YPD solid media to obtain a single colony. Three random colonies of similar size (three biological replicates) were inoculated in 5 ml YPD liquid media and grown overnight (12 hr) in a shaker at 200 rpm at 30°C. Cell density was measured at OD_600_ using a spectrophotometer. Cells were diluted to an O.D_600_ of 1.0 to achieve similar cellular concentrations. Serial dilution was performed in 1:10 proportion by pipetting 100 μl in 1 ml media. 2.5 μl of serially diluted samples were spotted on different YPD agar plates with exposure to environmental conditions. Images of the spot assay with uniform background brightness were acquired using a ChemiDoc XRS+ (BIO-RAD) instrument.

Growth rate assays were performed for the seven environmental conditions before and after propagation of the MA lines. Cells from single colonies were collected using 1 μl of 1 M sorbitol at t = 24/36/48 hr, depending on colony growth. Cell counts were obtained using a haemocytometer. Assuming the growth is exponential, the growth rate was estimated using the formula *N* *=* *N*_*0*_ x *e*^*rt*^ (*N* = Final cell count, *N*_*0 *_= Initial cell count, *r* = Growth rate/hour, *t* = time (hour) [7,27]. From the growth rate, the generation time (g) for the cells propagated under various environments was estimated using g = ln2/r hours. The number of generations for 24 hours was estimated using 24/g.

### Tetrad dissection and spore viability

Diploids were sporulated for ~ 3–5 days at 30°C on sporulation media (1% Potassium acetate, 0.1% Yeast extract, 0.05% Glucose). Tetrads were dissected using a tetrad-dissection microscope (Zeiss) on SC plate (0.7% Yeast nitrogen base, 0.09% Complete amino acid mix, 2% Glucose). The spores were grown for three days at 30°C in the incubator to determine viability.

### Mutation accumulation lines

Based on the initial growth rate estimation, 16 MA lines per environment were propagated for ~ 1000 generations (S1 Table). An average sized colony was selected randomly and streaked onto a new plate for propagation through single cell bottlenecks. The final bottlenecks at ~ 1000 generations were stored as glycerol stock. Out of 16 MA lines/environmental condition, colonies from 10 MA lines (final bottleneck) from each environment were sequenced on Illumina platform.

### DNA extraction and short read whole genome Illumina sequencing

MasterPure Yeast DNA Purification Kit from Lucigen was used to isolate genomic DNA from 5 ml cultures of the MA lines following the manufacturer’s protocol. The quality of the DNA extracted was quantified using Qubit. The genomic DNA was sequenced on Illumina Hi-Seq 4000 (Macrogen) and NovaSeq 6000 (Clevergene) platform.

### Processing of Illumina short read data

Quality control checks on Illumina raw reads were performed using FastQC. Based on sequence quality reports, the reads were processed using Trimmomatic (version 0.39) [83] to improve the read quality and to filter the adapter sequence for downstream analysis. Quality filtered paired-end reads were aligned to the S288c reference genome (version 64-1-1, 2011) using Bowtie 2 (version 2.3.5.1) [84] with default parameters. The aligned reads were sorted and indexed using samtools (version 1.10). Duplicate reads were removed using Picard tools.

### Analysis of SNMs, small indels

Freebayes (version 1.3.2) [85] was used to call SNMs and small indels. Mutational events shared by parents or with other lines were filtered out. Mutations called from the repetitive regions were also eliminated. A final set of mutations with GQ (Genotype Quality)>= 30, DP (Read Depth)>= 40, allelic ratio <= 0.1 or >= 0.9 for homozygous and > 0.4 and < 0.6 for heterozygous mutations, and bi-allelic were considered as de-novo mutations. SNM rate and indel rate per nucleotide per generation for an MA line was calculated by dividing the total number of mutations observed in an MA line by the number of generations and the genome size (24.04 Mb).

### Analysis of numerical aneuploidy

Whole chromosomal losses/gains and large segmental deletions/duplications were determined based on the sequence coverage analysis for the entire genome with a bin size of 5 kb [30,32]. Chromosomes/chromosomal segments where the read counts deviate from the median were verified using S288c allele frequency data. Lack of aneuploidy gives approximately 0.5 allele frequency, while the presence of aneuploidy causes a deviation in the S288c allele frequency from 0.5. The whole chromosome gain/loss rate per generation for an MA line was measured by dividing the number of gain/loss events observed at the final bottleneck by the total number of generations.

### Analysis of LOH events

SNP’s were called using GATK HaplotypeCaller (version 4.1.9.0). Heterozygous SNP positions in the parental genome (S288c/YJM789) that were genotyped in all MA lines for a given environment were used for the analysis. This corresponded to ~ 52,000 heterozygous SNPs in each environment (S4 Table). A bioinformatic pipeline written in R was used to detect LOH events as follows: If the genotype of the SNPs supporting LOH were homozygous for the S288c (0/0) reference genome, we annotated such LOH events as S288c fixed. If the SNP genotype is homozygous for the YJM789 genome (1/1), we annotated them as YJM789 fixed events. Further, LOH tracts supported by at least three SNPs fixed to either YJM789 or S288c were only considered. LOH tracts having identical start and end positions in all MA lines for a given environment were excluded to reduce the error rate for LOH detection. LOH tract sizes were estimated as described in [30]. To generate LOH maps, the yeast genome was partitioned into 1 bp bins and the LOH count was plotted for each bin.

The LOH data were also represented as histograms and density plots. For histograms, the LOH data was divided into bins, where the height of each bar represents the frequency of data points in that bin. The density plots used a kernel density estimation method to smooth the distribution, providing a clearer picture of the overall distribution without the discrete jumps seen in a histogram. The data was normalized to give a relative comparison, where the total area under the curve is always 1 (Y axis). A logarithmic scale (X axis) was used to visualize the frequency of smaller LOH events to much larger ones. The peaks in the density plot curve highlight LOH lengths that are more frequent, while valleys indicate lengths that occur less often. An unimodal curve (one peak) suggests that LOH lengths cluster around a specific range. A multimodal curve (multiple peaks) suggests distinct subgroups of LOH lengths.

LOH events were also annotated as interstitial and terminal. LOH events were considered terminal if at least one of the SNP markers was within 20 kb of the telomere and the other markers were outside the 20 kb window [32]. All other LOH events were annotated as interstitial. LOH events were classified based on probable mechanism (recombination or deletion) as described in [6] and [32]. Briefly, normalized coverage for S288c and YJM789 alleles were obtained by dividing allele specific coverage for the S288c and YJM789 alleles with the average coverage of S288c plus YJM789 alleles. If the normalized allele-specific coverage values for one of the alleles were between 0.4 and 0.6 and the other allele ≤ 0.1, they were classified as deletions. The rest were classified as potentially arising from recombination events.

### High molecular weight genomic DNA isolation and structural variant analysis from long read sequence data (Nanopore)

High molecular weight DNA was isolated from 11 samples (One parental, five control and five blue light exposed MA lines) using MasterPure Yeast DNA purification kit following manufacturers protocol (Biosearch Technologies) and as described in [86]. The DNA concentration was evaluated using a Qubit flurometer, and the quality was determined using Nanodrop Spectrophotometer. The samples were sequenced on Nanopore PromethION system, using PromethION flow cell (ONT, FLO-PRO114M) at Genotypic (India). Nanoplot (version 1.30.1) was used to generate QC graphs to determine sequence quality. Adapters were removed using Porechop (0.2.4) with default parameters. The processed reads were aligned to S288c reference genome (version 64-1-1, 2011) using minmap2 (version 2.28-r1209) aligner with the –ax lr:hq preset for Nanopore Q20 genomic reads. Samtool was used for file format conversion (SAM to BAM), coordinate sorting and indexing. The genomic variants were called using Sniffles2 (version 2.2), a structural variant caller specifically designed for long-read sequencing reads (Oxford nanopore). Structural variants were called from the ancestral strain (S288c/YJM789) and five MA lines each from YPD and blue light conditions. Structural variants shared with the ancestral strain or having identical start and end points among all the MA lines were removed from the analysis. Additional filters included removal of imprecise structural variants, and structural variants with strand bias, < 50 bp in length, with read support < 10 and quality < 30 during downstream analysis.

### Acellular alkaline comet assay

Comet assay was performed using a protocol adapted from [87]. Briefly, yeast cell pellets (S288c/YJM789) were resuspended in 1 ml of Zymolase buffer (20T Zymolase, 1 ml S buffer) and incubated at 30°C for 60 min to obtain spheroplasts. Spheroplasts were resuspended in 1.5% LMA (Low melting agarose w/v in S buffer) at 35°C. 80 μl of the suspension were spread per glass slide and allowed to solidify. The glass slides were submerged in lysis buffer (30 mM NaOH, 1 M NaCl, 0.05% (w/v) lauroylsarcosine, 50 mM EDTA, 10 mM Tris, adjusted to pH 10) for 60 min at 4°C. Excess lysis buffer was drained, and the slide was submerged in neutralization buffer (10 mM Tris-HCl, adjusted to pH 7.4). Genomic DNA embedded in LMA were treated as follows: For the control treatment, slides were kept in dark for 36 hr at 30°C in the incubator; for blue light exposure, slides were exposed to blue light at low intensity (500 μmol·m−2·s−1) for 36 hr at 30°C in the incubator. After treatment, slides were submerged in electrophoresis buffer (30 mM NaOH, 10 mM EDTA, 10 mM Tris-HCl, adjusted to pH 10) for 20 min at 4°C. Electric field (0.7V/cm) was applied for 10 min in an electrophoresis setup. Excess electrophoresis buffer was drained, and the microgel was neutralized by submerging it in neutralization buffer (10 mM Tris-HCl, adjusted to pH 7.4) for 10 min. The slides were washed with 76% and 96% ethanol for 10 minutes each and air dried at room temperature. The slides were stained with 5 μg/ml of Propidium iodide and analysed under a fluorescence microscope. 100 comets were analysed for each treatment using OpenComet v1.3.1. Three biological replicates were analysed for control and blue light exposure.

The extent of the DNA damage was quantified using % Tail DNA, Tail Moment and Olive Tail Moment. % Tail DNA represents the percentage of the total DNA present in the tail. Higher percentage of tail DNA indicates more fragmented DNA is present in the tail, which implies more damage is induced by the mutagen. The Tail Moment (Tail length × %Tail DNA) is the combined measure of tail length (distance migrated by the fragmented DNA) and DNA intensity (Percentage of fragmented DNA present in the tail) which gives the overall impact of the mutagen on the genome. Olive Tail Moment picks up variations in DNA distribution within the tail. It is calculated as the length in terms of distance between the centre of the head and tail of the comet times the percentage of Tail DNA.

### Gene expression analysis by RT-qPCR

To assess gene expression post-blue light exposure, cells were collected after growing on solid YPD media for 36 hr from control and samples irradiated with blue light (λ = 470 nm) at low (500 μmol·m−2·s−1) and high (920 μmol·m−2·s−1) intensity. Total RNA was extracted using RiboPure-Yeast Kit (Invitrogen; LOT:2835867). Integrity of RNA was verified by gel electrophoresis to check for the presence of two distinct bands of 18s and 25s rRNA subunits with minimal to no smearing. Reverse transcription was performed, using Verso cDNA synthesis kit for RT-PCR (Invitrogen; LOT: 2903615; Cat: AB1453A) with random hexamers and oligo dT, using 500 ng of RNA. Real-time PCR was performed for candidate genes (*POL3*, *REV3*, *RAD7*, *RAD26*, *RAD30*, *RAD51*, *YAP1*, *PIF1*, *POL4* and *MSH2*). Real-time PCR reactions were run on Biorad CFX connect, using TB Green Premix Ex Taq II (Takara; Cat: RR820A) using primers shown in S11 Table. Expression levels of candidate genes were normalized against *GAPDH* gene expression (House-keeping gene) and quantified by calculating 2^−ΔΔCt^. A total of three biological replicates were analysed.

### Statistical tests

The Wilcoxon rank-sum test was used for comparisons involving multiple tests, followed by Bonferroni correction to adjust for multiple testing. For single-sample comparisons, the Wilcoxon signed-rank test was applied without correction. In addition, Chi-Square tests were used for categorical data analysis, with Bonferroni correction applied to account for multiple comparisons where applicable. Statistical significance was set at p < 0.05 (*), p < 0.01 (**), p < 0.001 (***), p < 0.0001 (****) and all analyses were performed using R. ns indicates not significant (p > 0.05).

## Supporting information

S1 Fig Environmental conditions used for MA lines. **A)** Spot assay of the parent S288c/YJM789 hybrid to determine viability at the final concentration/ condition of each environment before starting the MA lines. **B)** Boxplot showing growth rate under the seven environmental conditions before and after the MA experiments. Triangles indicate the average growth rate. Statistical differences were assessed by Wilcoxon rank-sum test (* p < 0.05, ** p < 0.01, *** p < 0.001). ns indicates not significant. **C)** Boxplot showing spore viability after ~1000 generations under each environmental condition. Triangles indicate the average spore viability.
(PDF)

S2 Fig MA lines from seven environments patched on YPL (Yeast extract-1%, Peptone-2%, Lactate-2%) after ~1000 generations. (PDF)

S3 Fig A) Distribution of heterozygous SNPs in the S288c/YJM789 (S/Y) parent hybrid. Black dots indicate the centromere position. Red lines show the heterozygous SNP positions along the chromosome. **B) Mean LOH rate per generation across environments. C,D) LOH counts across environments supported by C) ≥ 5 SNPs, D) ≥ 10SNPs.** Statistical differences in the LOH rate and count, between an environment and the control (YPD) were assessed by Wilcoxon rank-sum test (** p < 0.01) followed by Bonferroni correction.
(PDF)

S4 Fig A) Distribution of LOH events pooled from seven environments along with SNP density. LOH counts are shown along the chromosome with 1 bp bin size. SNP density is shown with 100 bp bin size. The black dot marks the centromere position. **B) Distribution of terminal LOH tracts pooled from seven environments across all sixteen chromosomes.** The black dots represent the centromere. LOH counts are shown along the chromosome with 1 bp bin size. **C) Distribution of interstitial LOH tracts pooled from seven environments across all sixteen chromosomes.** The black dots represent the centromere. LOH counts are shown along the chromosome with 1 bp bin size.
(PDF)

S5 Fig Histogram showing the distance of the LOH events from the A) Centromere and B) Telomere in seven different environments. Vertical dotted lines show the mean distance.
(PDF)

S6 Fig Distribution of LOH tract size for all the environments. A, B) Density plot of the LOH tract sizes for all the environments. Vertical dotted lines show the median LOH tract size for each environment. LOH tract sizes in each environment significantly differed from the YPD control (p < 0.0001). LOH tract size for CR were significantly shorter than blue light and H_2_O_2_ (p < 0.0001). Statistical significance was assessed by Wilcoxon rank-sum test followed by Bonferroni correction. **C) Histogram represents the distribution of LOH counts across different categories of LOH tract sizes.** Total LOH counts are shown for short tracts (< 1000 bp), medium tracts (1000 – 10,000 bp), long tracts (10,000 – 100,000 bp), and super long tracts (> 100,000 bp). The vertical dotted lines represent the mean LOH tract size.
(PDF)

S7 Fig Sanger sequencing analysis of 7 SNMs and 3 small indels from MA lines. The line number, mutation (SNM/small indel) and the chromosomal locations are shown. Highlighted regions in the chromatograms show the mutations.
(PDF)

S8 Fig Read coverage and allele frequency plot of 10 MA lines from each environment: A) Rate of whole chromosomal gain/loss across the seven environments from Illumina short read data. Read coverage and allele frequency plots are shown for **B)** YPD, **C)** Ethanol, **D)** NaCl, **E)** High temperature, **F)** calorie restriction, **G)** H_2_O_2_, **H)** Blue light. For the read coverage plots, colored dots show read counts in 5 kb bin sizes. The median and 2x median read counts are represented by the horizontal, blue and red dotted lines respectively. For the allele frequency plots, black dots show the frequency for the S288c alleles (percentage). Blue, red and green dotted lines show 50% (2n), 33% (1n), 66%(3n) S288c allele frequency. Vertical lines show the chromosomal boundaries. Black arrows in the coverage plots show the aneuploidy events.
(PDF)

S9 Fig Proportion of LOH events arising from recombination versus deletion events. (PDF)

S1 TableFinal set of environmental conditions used for propagating MA lines.For each condition, the number of bottlenecks was adjusted based on the initial growth rate to achieve ~1000 generations. Growth rates were estimated prior to starting the MA lines (initial growth rate) and at the end of the MA line propagation (final growth rate). The total number of generations propagated under a specific environment was estimated using the following formula: Total no. of generations = Total no. of bottlenecks × (Initial Generations per Bottleneck+Final Generations per bottleneck)/2. Here, the “Initial Generations per Bottleneck” represents the number of generations per bottleneck prior to MA line propagation, while the “Final Generations per Bottleneck” represents the corresponding number at the final bottleneck.(PDF)

S2 Table Spore viability of S288c/YJM789 hybrid parent strain and five mutation accumulation lines from each environment. (XLSX)

S3 Table List of heterozygous SNPs (52,240) from the S288c/YJM789 parent used to call LOH tracts in the MA lines. SNP position on each chromosome is shown in base pairs. Genotype of 0 indicates the S288c allele and 1 indicates the YJM789 allele.
(XLSX)

S4 Table Read coverage and SNP distribution across 70 MA lines from seven different environmental conditions. (XLSX)

S5 Table LOH tracts accumulated in MA lines. A minimum of 3 SNPs supports the LOH tracts.
(XLSX)

S6 Table LOH tract profile across the seven environments. (XLSX)

S7 Table A) SNM rates for all MA lines propagated in seven different environmental conditions. **B) Small indel rate for all MA lines propagated in seven different environmental conditions. C) Spectrum of single nucleotide mutations in all seven environments.** These include transition and transversion events covering all six types of SNMs. The transition (Ts) to transversion (Tv) ratio and AT/GC bias (GC > AT/AT > GC) are also shown.
(XLSX)

S8 Table Chromosome gain/loss rate across seven different environments. (XLSX)

S9 Table Structural variants called in five MA lines propagated on YPD and blue light exposure. (XLSX)

S10 Table Fold changes in gene expression (2^−∆∆ Ct^) in three independent biological replicates at high and low intensity blue light exposure. (XLSX)

S11 Table Primers for RT-qPCR to analyze the expression of candidate genes under blue light exposure. (PDF)
