# Supplementary material for: Loss of Heterozygosity associated with ubiquitous environments in yeast
Source: PLoS Genet. 2025 May 12;21(5):e1011692. doi: 10.1371/journal.pgen.1011692 (PMC12068580; doi:10.1371/journal.pgen.1011692)
Supplement: S1 Fig — A) Spot assay of the parent S288c/YJM789 hybrid to determine viability at the final concentration/ condition of each environment before starting the MA lines. B) Boxplot showing growth rate under the seven environmental conditions before and after the MA experiments. Triangles indicate the average growth rate. Statistical differences were assessed by Wilcoxon rank-sum test (* p < 0.05, ** p < 0.01, *** p < 0.001). ns indicates not significant. C) Boxplot showing spore viability after ~1000 generations under each environmental condition. Triangles indicate the average spore viability. (PDF) [file pgen.1011692.s001.pdf]

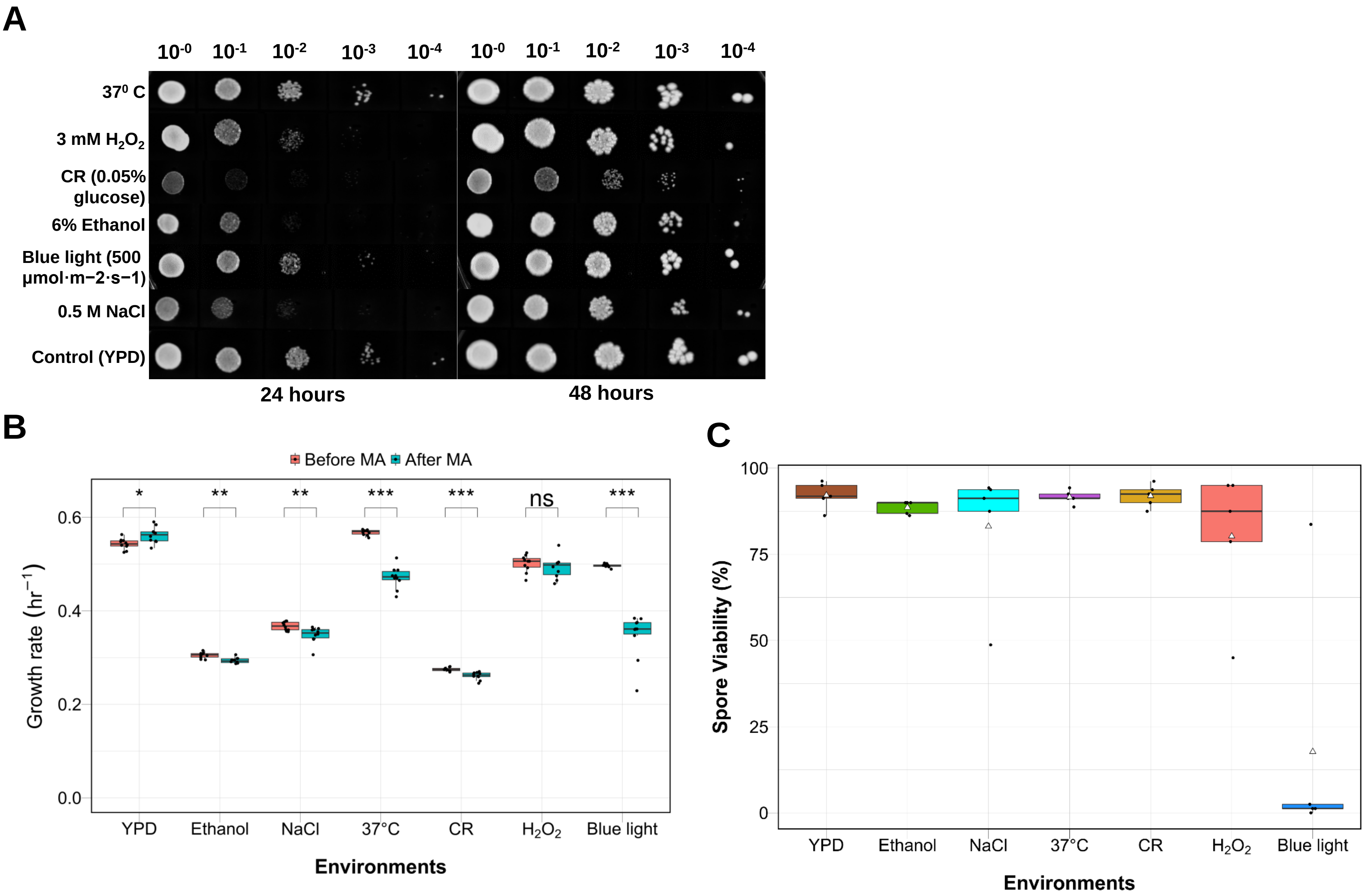

S1 Fig. Environmental conditions used for MA lines. A) Spot assay of the parent S288c/YJM789 hybrid to determine viability at the final concentration / condition of each environment before starting the MA lines. **B)** Boxplot showing growth rate under the seven environmental conditions before and after the MA experiments. Triangles indicate the average growth rate. Statistical differences were assessed by Wilcoxon rank-sum test (* $p < 0.05$, ** $p < 0.01$, *** $p < 0.001$). ns indicates not significant. **C)** Boxplot showing spore viability after ~1000 generations under each environmental condition. Triangles indicate the average spore viability.
