# Supplementary material for: Loss of Heterozygosity associated with ubiquitous environments in yeast
Source: PLoS Genet. 2025 May 12;21(5):e1011692. doi: 10.1371/journal.pgen.1011692 (PMC12068580; doi:10.1371/journal.pgen.1011692)
Supplement: S2 Fig — (PDF) [file pgen.1011692.s002.pdf]

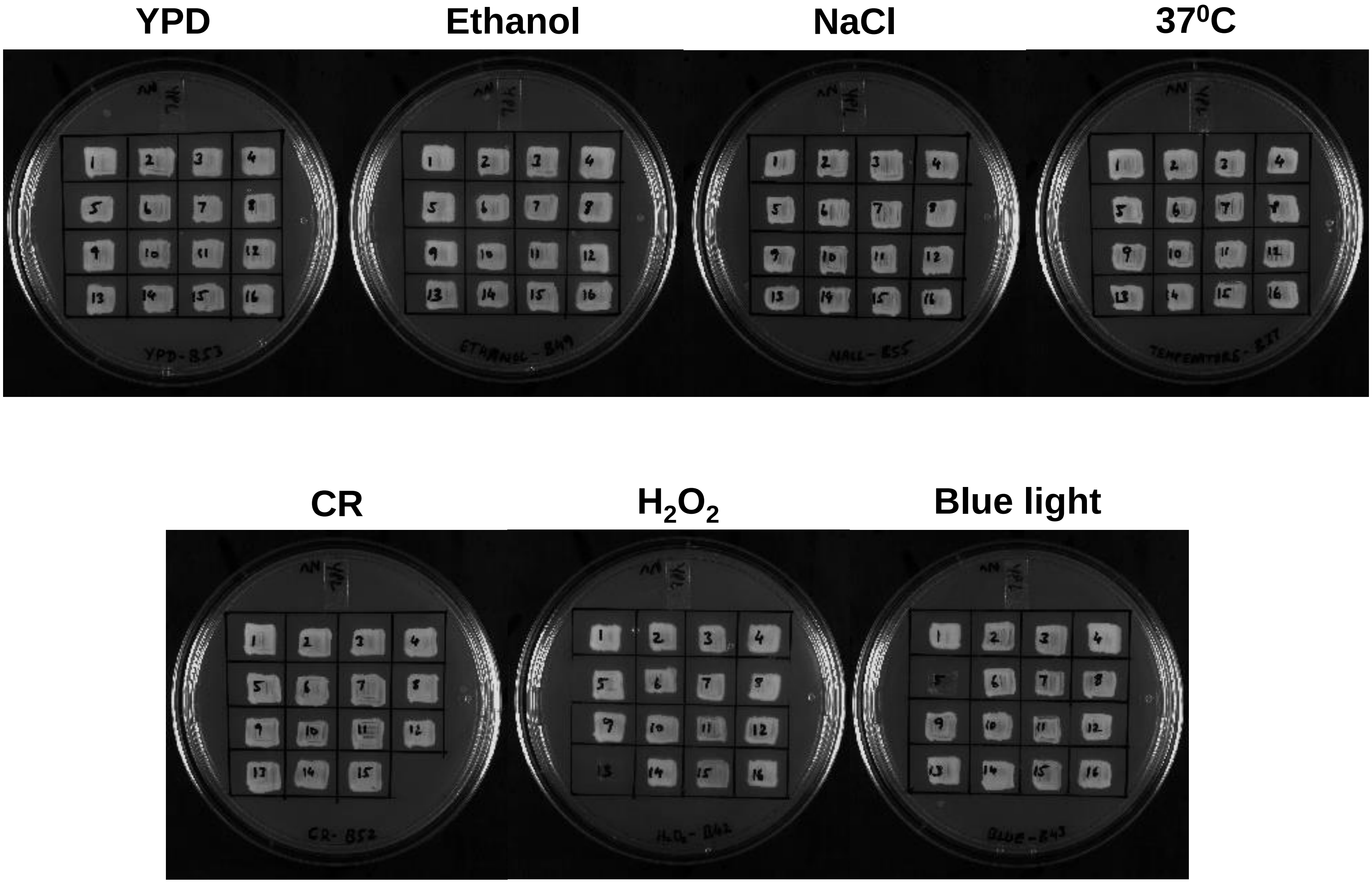

S2 Fig. MA lines from seven environments patched on YPL (Yeast extract-1%, Peptone-2%, Lactate-2%) after ~1000 generations.
