# Supplementary material for: Loss of Heterozygosity associated with ubiquitous environments in yeast
Source: PLoS Genet. 2025 May 12;21(5):e1011692. doi: 10.1371/journal.pgen.1011692 (PMC12068580; doi:10.1371/journal.pgen.1011692)
Supplement: S3 Fig — Black dots indicate the centromere position. Red lines show the heterozygous SNP positions along the chromosome. B) Mean LOH rate per generation across environments. C,D) LOH counts across environments supported by C) ≥ 5 SNPs, D) ≥ 10SNPs. Statistical differences in the LOH rate and count, between an environment and the control (YPD) were assessed by Wilcoxon rank-sum test (** p < 0.01) followed by Bonferroni correction. (PDF) [file pgen.1011692.s003.pdf]

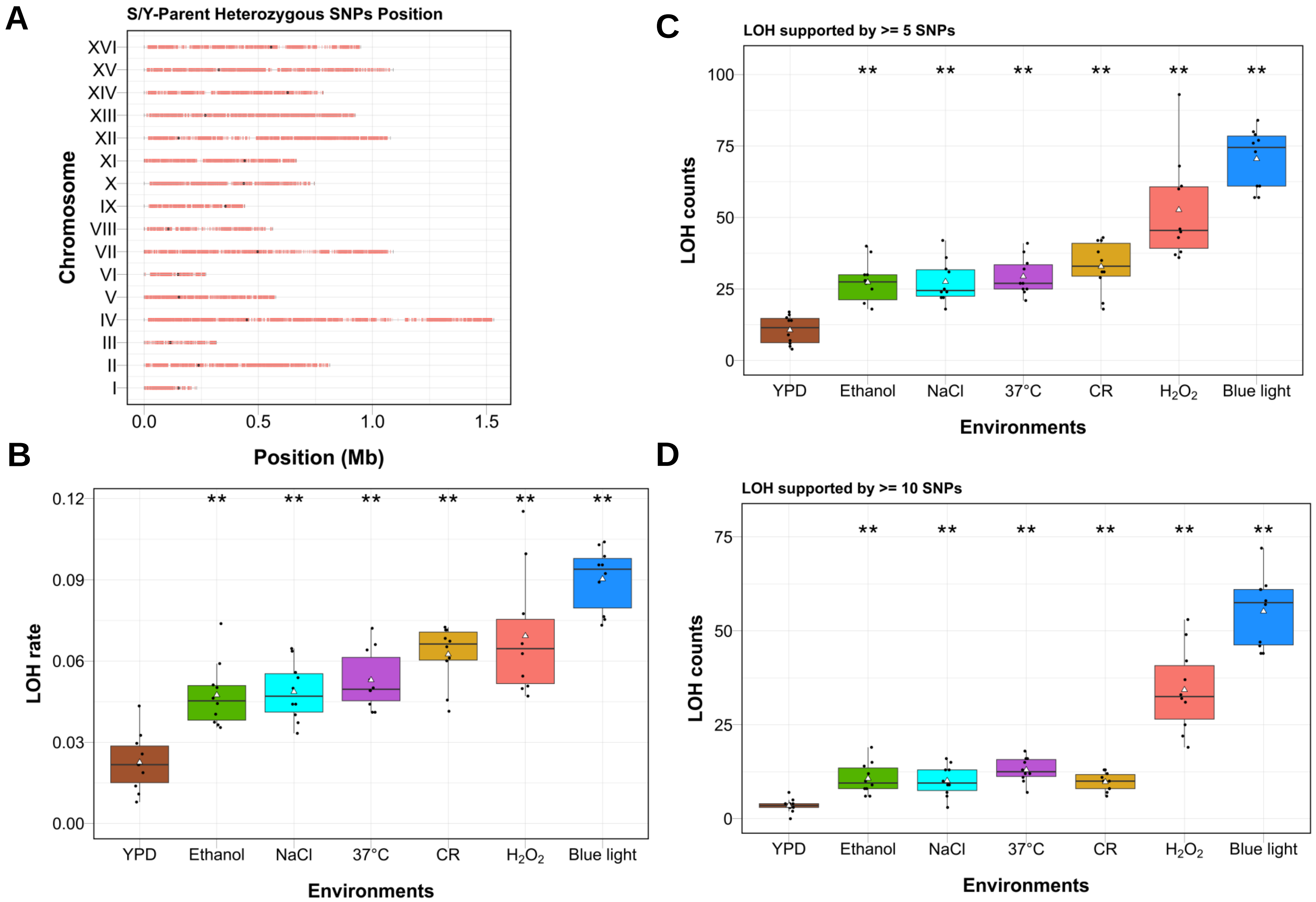

S3 Fig. A) Distribution of heterozygous SNPs in the S288c/YJM789 (S/Y) parent hybrid. Black dots indicate the centromere position. Red lines show the heterozygous SNP positions along the chromosome. **B) Mean LOH rate per generation across environments. C,D) LOH counts across environments supported by C) ≥ 5 SNPs, D) ≥ 10SNPs.** Statistical differences in the LOH rate and count, between an environment and the control (YPD) were assessed by Wilcoxon rank-sum test (** $p < 0.01$) followed by Bonferroni correction.
