# Supplementary material for: Loss of Heterozygosity associated with ubiquitous environments in yeast
Source: PLoS Genet. 2025 May 12;21(5):e1011692. doi: 10.1371/journal.pgen.1011692 (PMC12068580; doi:10.1371/journal.pgen.1011692)
Supplement: S4 Fig — LOH counts are shown along the chromosome with 1 bp bin size. SNP density is shown with 100 bp bin size. The black dot marks the centromere position. B) Distribution of terminal LOH tracts pooled from seven environments across all sixteen chromosomes. The black dots represent the centromere. LOH counts are shown along the chromosome with 1 bp bin size. C) Distribution of interstitial LOH tracts pooled from seven environments across all sixteen chromosomes. The black dots represent the centromere. LOH counts are shown along the chromosome with 1 bp bin size. (PDF) [file pgen.1011692.s004.pdf]

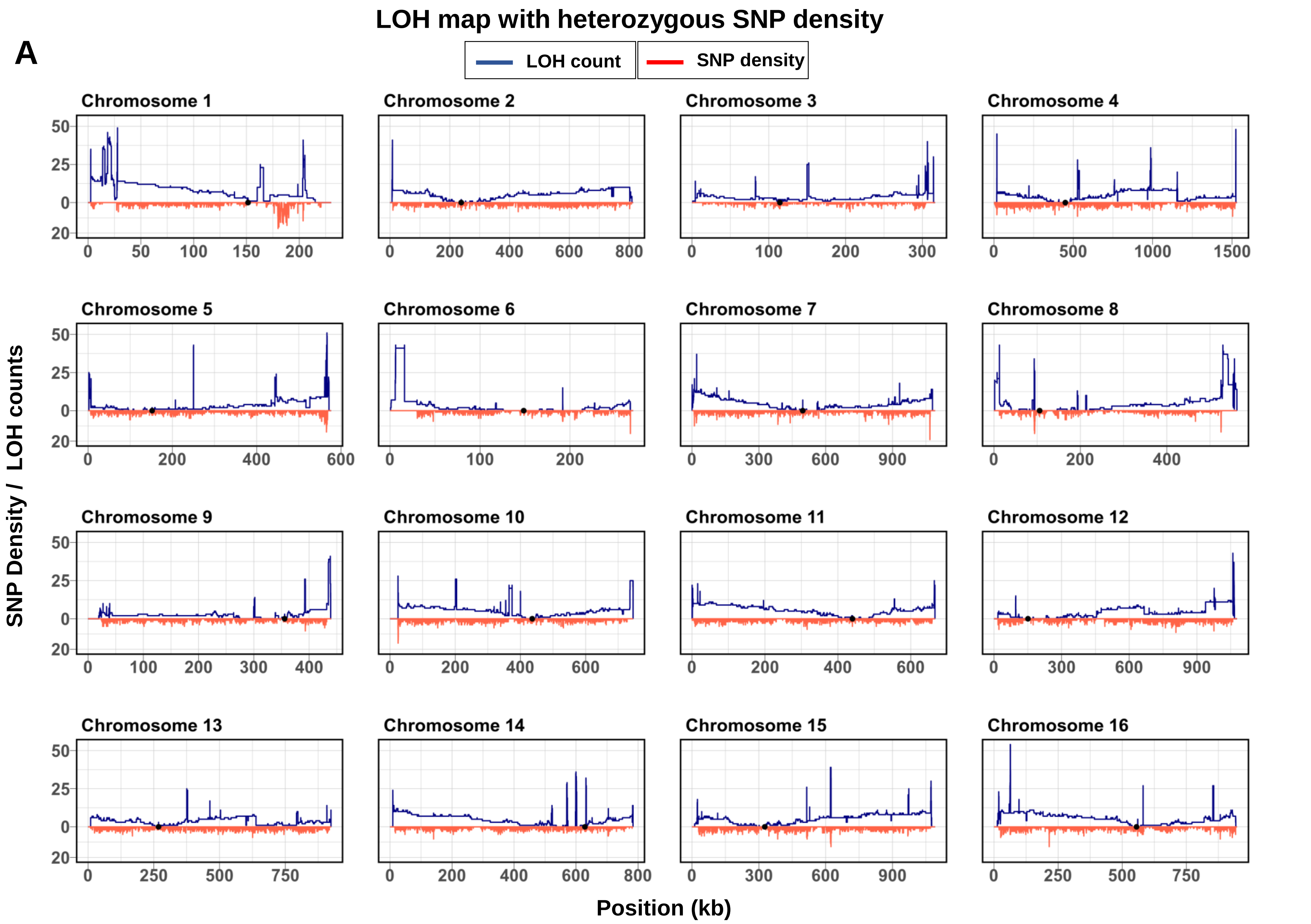

S4A Fig. Distribution of LOH events pooled from seven environments along with SNP density. LOH counts are shown along the chromosome with 1 bp bin size. SNP density is shown with 100 bp bin size. The black dot marks the centromere position.

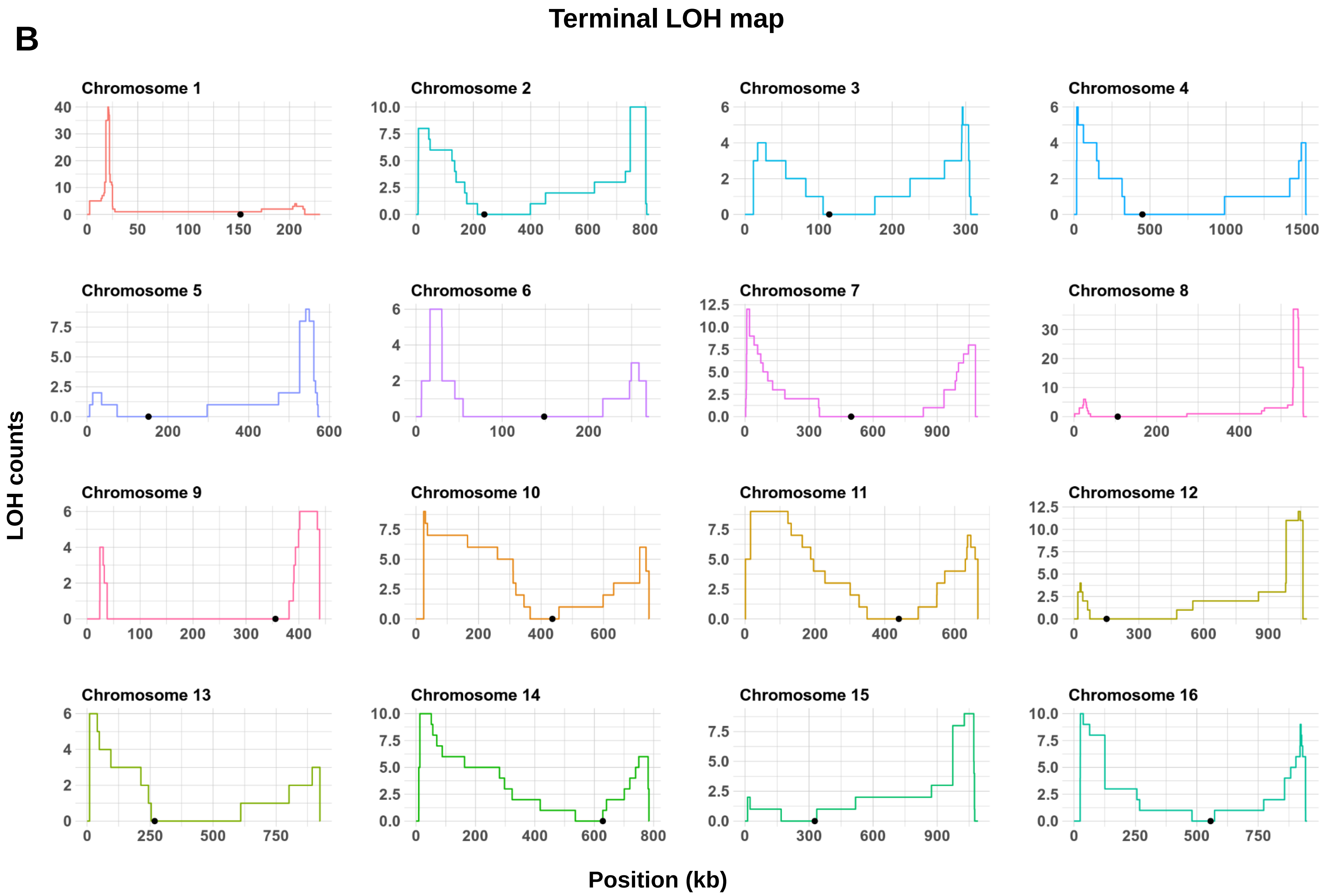

S4B Fig. Distribution of terminal LOH tracts pooled from seven environments across all sixteen chromosomes. The black dots represent the centromere. LOH counts are shown along the chromosome with 1 bp bin size.

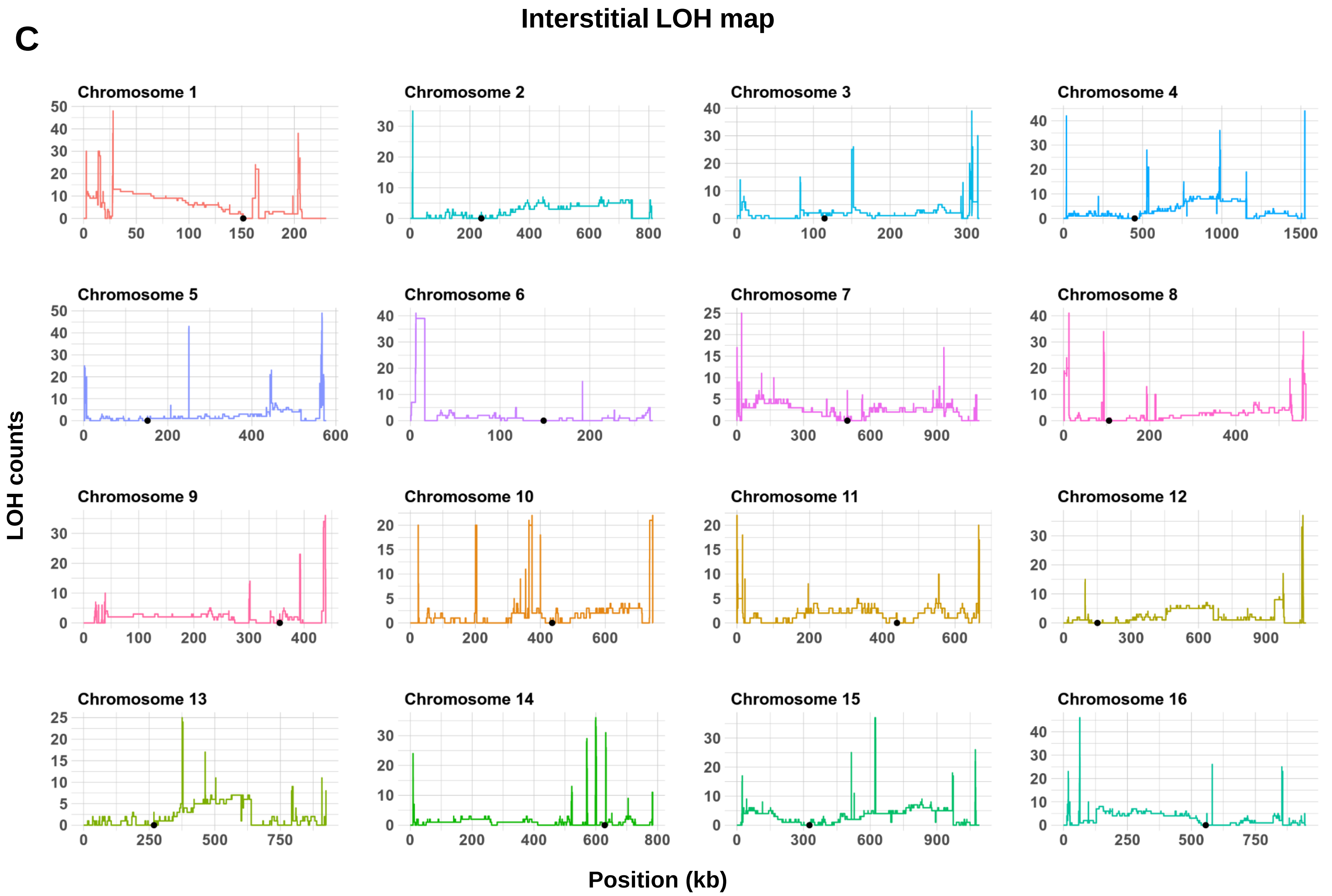

S4C Fig. Distribution of Interstitial LOH tracts pooled from seven environments across all sixteen chromosomes. The black dots represent the centromere. LOH counts are shown along the chromosome with 1 bp bin size.
