# Supplementary material for: Loss of Heterozygosity associated with ubiquitous environments in yeast
Source: PLoS Genet. 2025 May 12;21(5):e1011692. doi: 10.1371/journal.pgen.1011692 (PMC12068580; doi:10.1371/journal.pgen.1011692)
Supplement: S5 Fig — Vertical dotted lines show the mean distance. (PDF) [file pgen.1011692.s005.pdf]

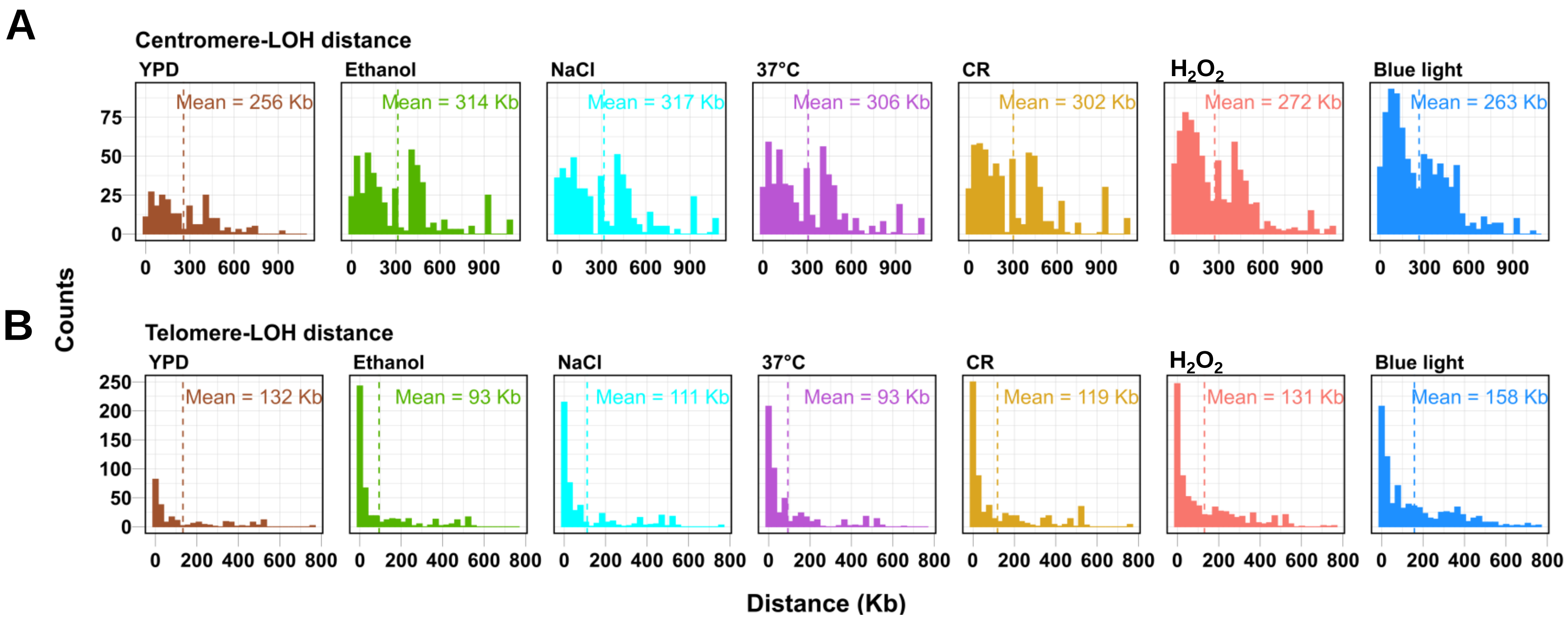

S5 Fig. Histogram showing the distance of the LOH events from the A) Centromere and B) Telomere in seven different environments. Vertical dotted lines show the mean distance.
