# Supplementary material for: Loss of Heterozygosity associated with ubiquitous environments in yeast
Source: PLoS Genet. 2025 May 12;21(5):e1011692. doi: 10.1371/journal.pgen.1011692 (PMC12068580; doi:10.1371/journal.pgen.1011692)
Supplement: S6 Fig — Vertical dotted lines show the median LOH tract size for each environment. LOH tract sizes in each environment significantly differed from the YPD control (p < 0.0001). LOH tract size for CR were significantly shorter than blue light and H2O2 (p < 0.0001). Statistical significance was assessed by Wilcoxon rank-sum test followed by Bonferroni correction. C) Histogram represents the distribution of LOH counts across different categories of LOH tract sizes. Total LOH counts are shown for short tracts (< 1000 bp), medium tracts (1000 – 10,000 bp), long tracts (10,000 – 100,000 bp), and super long tracts (> 100,000 bp). The vertical dotted lines represent the mean LOH tract size. (PDF) [file pgen.1011692.s006.pdf]

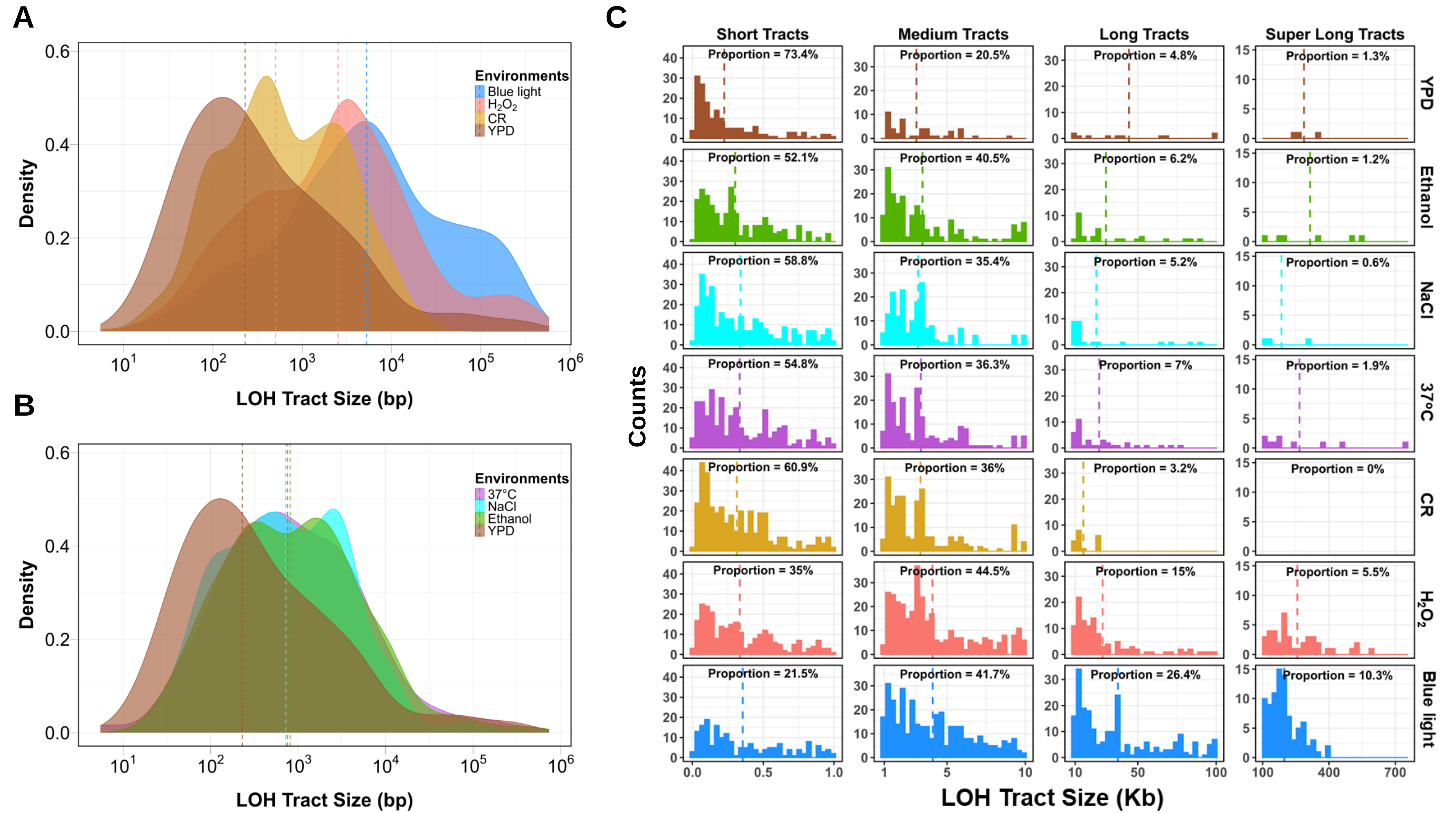

S6 Fig. Distribution of LOH tract size for all the environments. A, B) Density plot of the LOH tract sizes for all the environments. Vertical dotted lines show the median LOH tract size for each environment. LOH tract sizes in each environment significantly differed from the YPD control ($p < 0.0001$). LOH tract size for CR were significantly shorter than blue light and H_2O_2 ($p < 0.0001$). Statistical significance was assessed by Wilcoxon rank-sum test followed by Bonferroni correction. **C) Histogram represents the distribution of LOH counts across different categories of LOH tract sizes.** Total LOH counts are shown for short tracts (< 1000 bp), medium tracts (1000 – 10,000 bp), long tracts (10,000 – 100,000 bp), and super long tracts (> 100,000 bp). The vertical dotted lines represent the mean LOH tract size.
