# Supplementary material for: Loss of Heterozygosity associated with ubiquitous environments in yeast
Source: PLoS Genet. 2025 May 12;21(5):e1011692. doi: 10.1371/journal.pgen.1011692 (PMC12068580; doi:10.1371/journal.pgen.1011692)
Supplement: S7 Fig — The line number, mutation (SNM/small indel) and the chromosomal locations are shown. Highlighted regions in the chromatograms show the mutations. (PDF) [file pgen.1011692.s007.pdf]

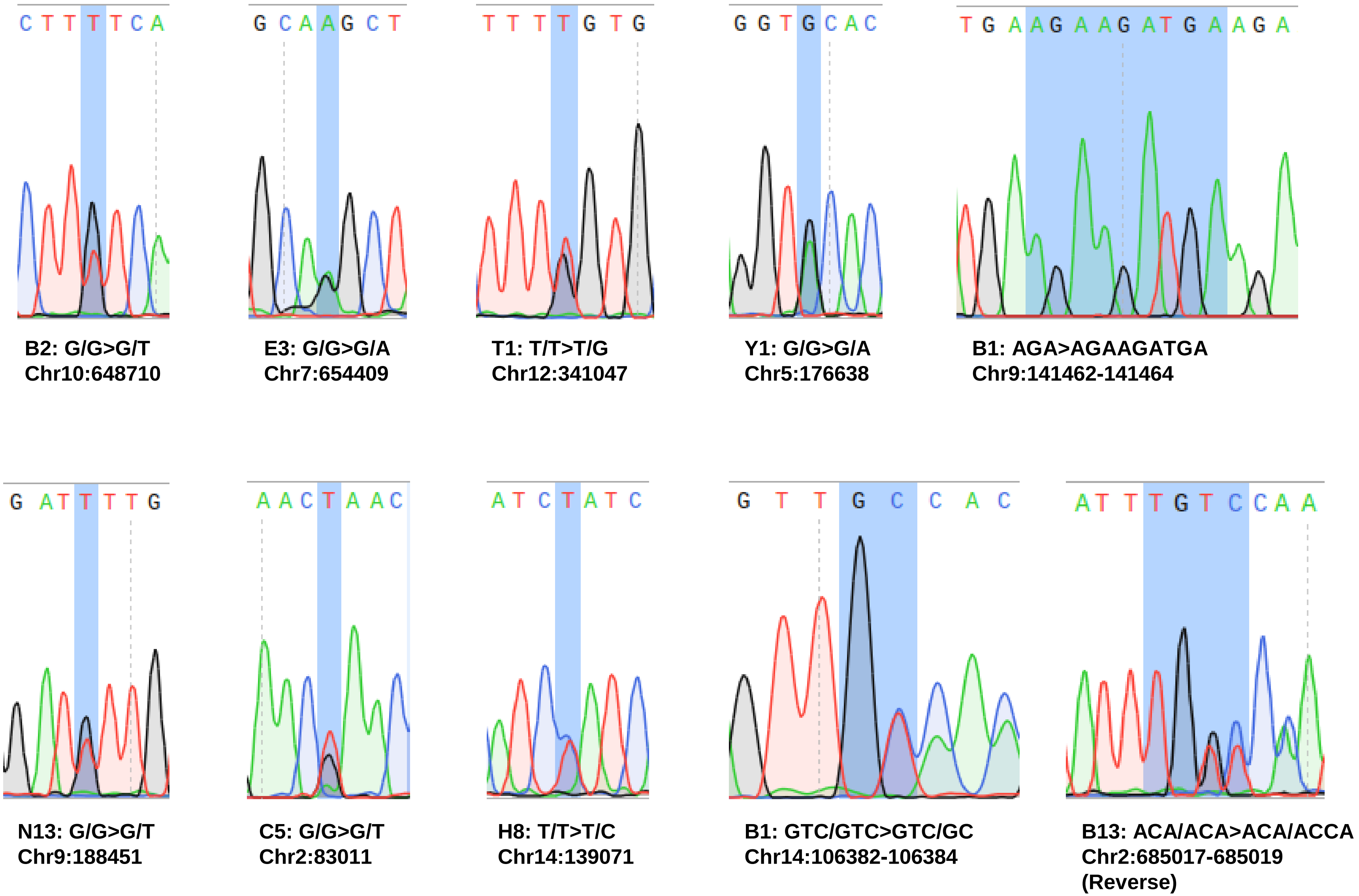

S7 Fig. Sanger sequencing analysis of 7 SNMs and 3 small indels from MA lines. The line number, mutation (SNM/small indel) and the chromosomal locations are shown. Highlighted regions in the chromatograms show the mutations.
