# Supplementary material for: Loss of Heterozygosity associated with ubiquitous environments in yeast
Source: PLoS Genet. 2025 May 12;21(5):e1011692. doi: 10.1371/journal.pgen.1011692 (PMC12068580; doi:10.1371/journal.pgen.1011692)

S8 Fig. Read coverage and allele frequency plot of 10 MA lines from each environment: **A)** Rate of whole chromosomal gain/loss across the seven environments from Illumina short read data. Read coverage and allele frequency plots are shown for **B)** YPD, **C)** Ethanol, **D)** NaCl, **E)** High temperature, **F)** calorie restriction, **G)** H_2O_2, **H)** Blue light. For the read coverage plots, colored dots show read counts in 5 kb bin sizes. The median and 2x median read counts are represented by the horizontal, blue and red dotted lines respectively. For the allele frequency plots, black dots show the frequency for the S288c alleles (percentage). Blue, red and green dotted lines show 50% (2n), 33% (1n), 66%(3n) S288c allele frequency. Vertical lines show the chromosomal boundaries. Black arrows in the coverage plots show the aneuploidy events.

A

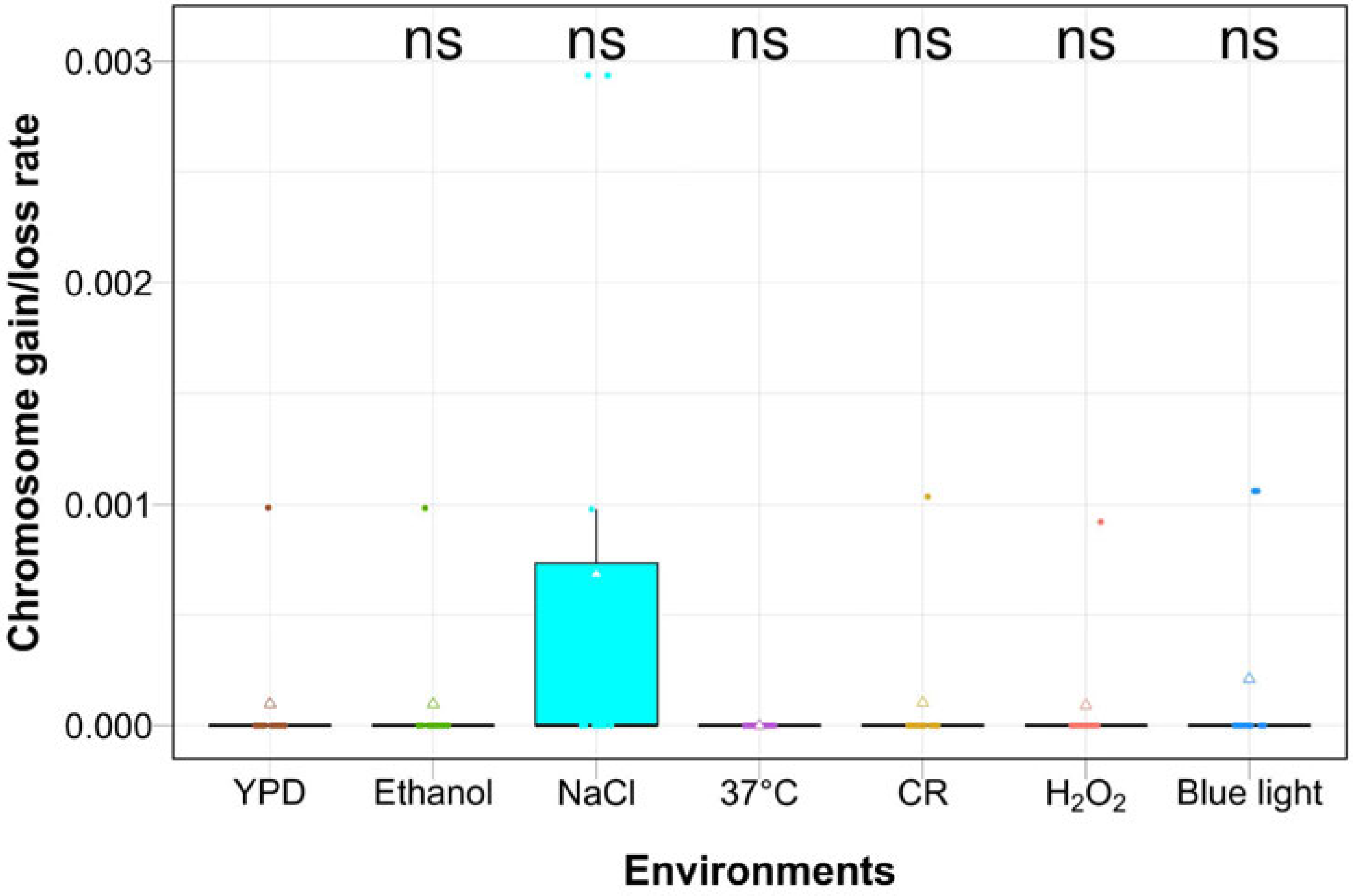
ns
ns
ns
ns
ns
ns
0.003
0.002
0.001
0.000
Chromosome gain/loss rate
YPD
Ethanol
NaCl
37°C
CR
H2O2
Blue light
Environments

B

YPD – Aneuploidy profile

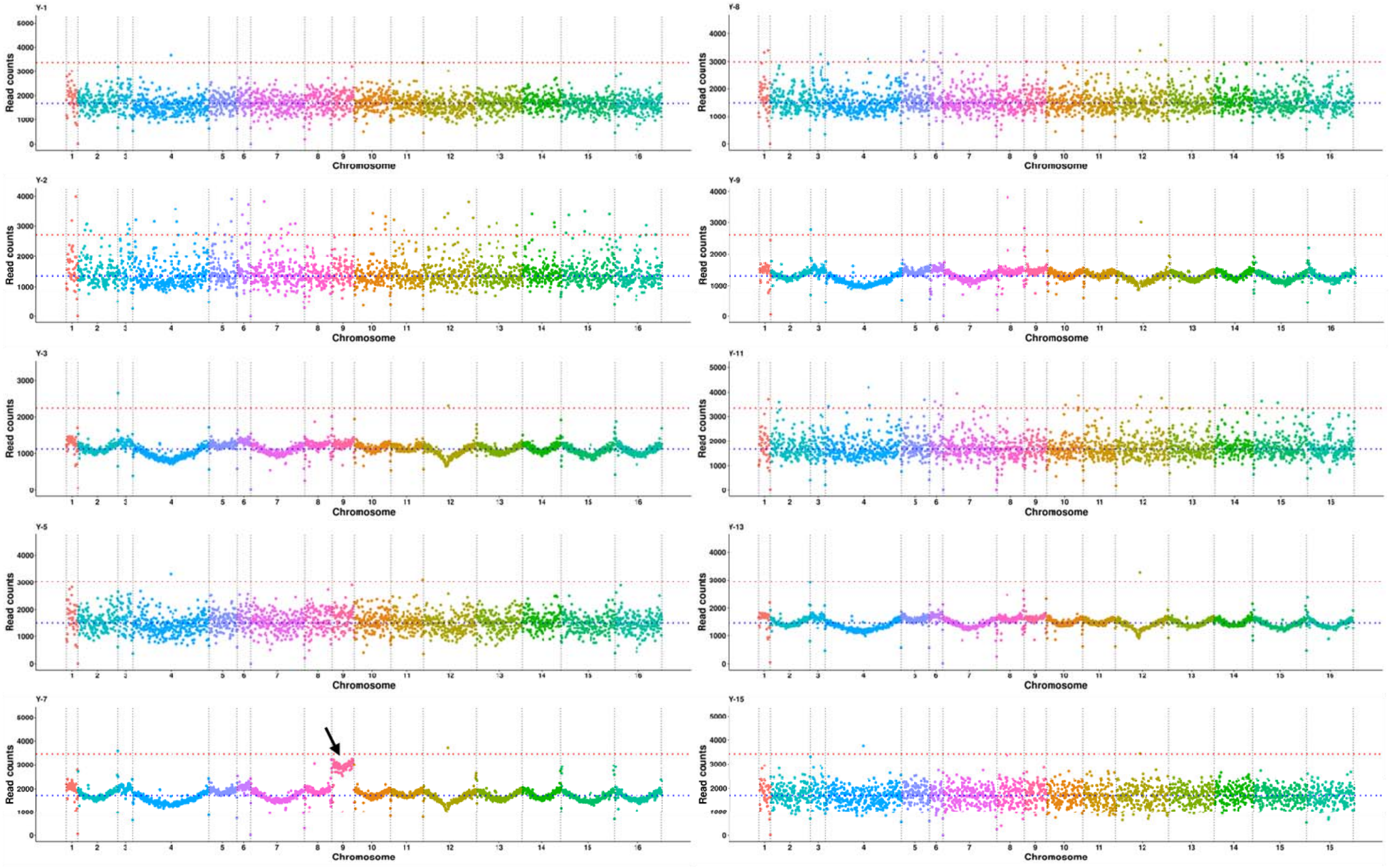

B

YPD – Aneuploidy profile

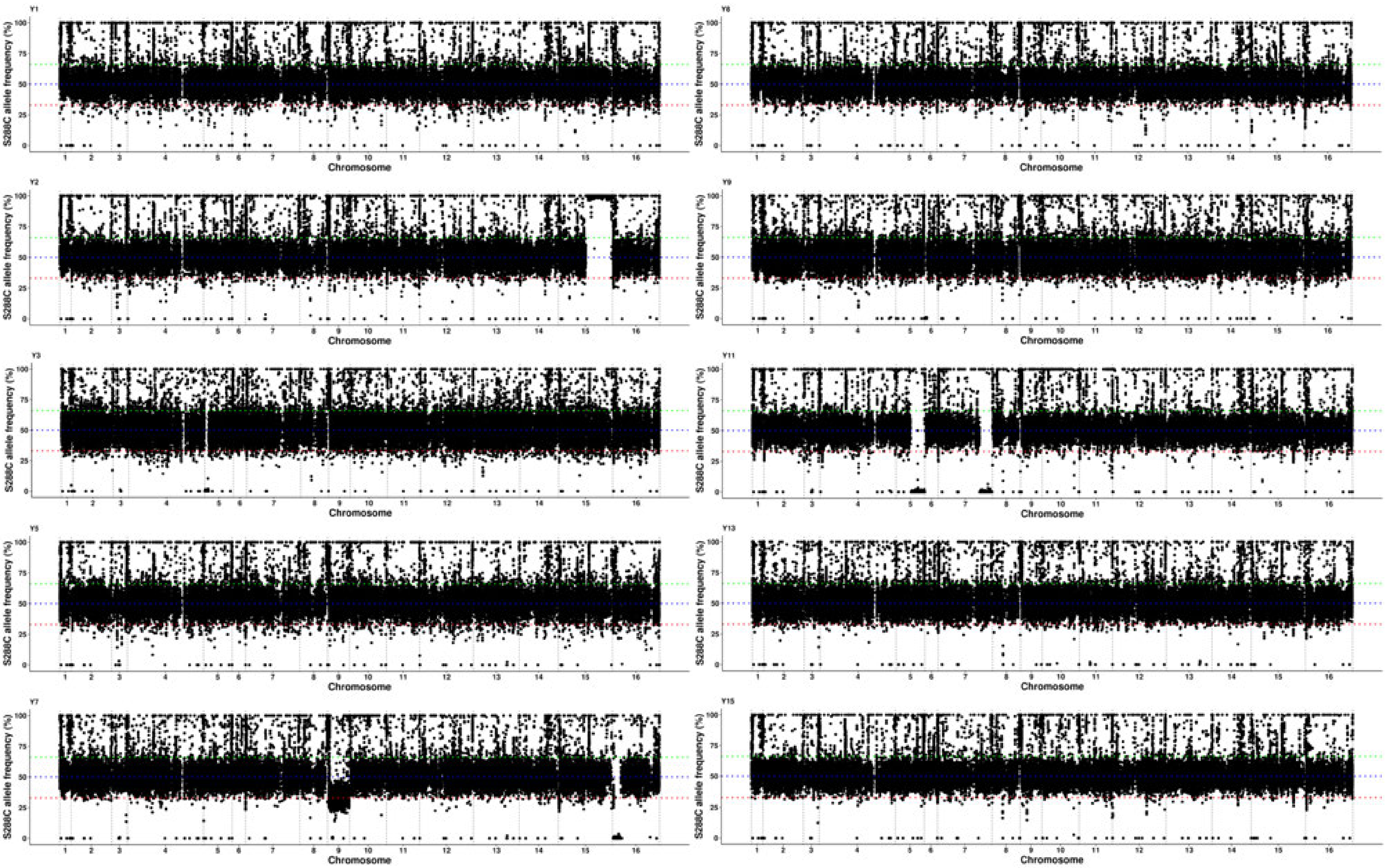

C

Ethanol – Aneuploidy profile

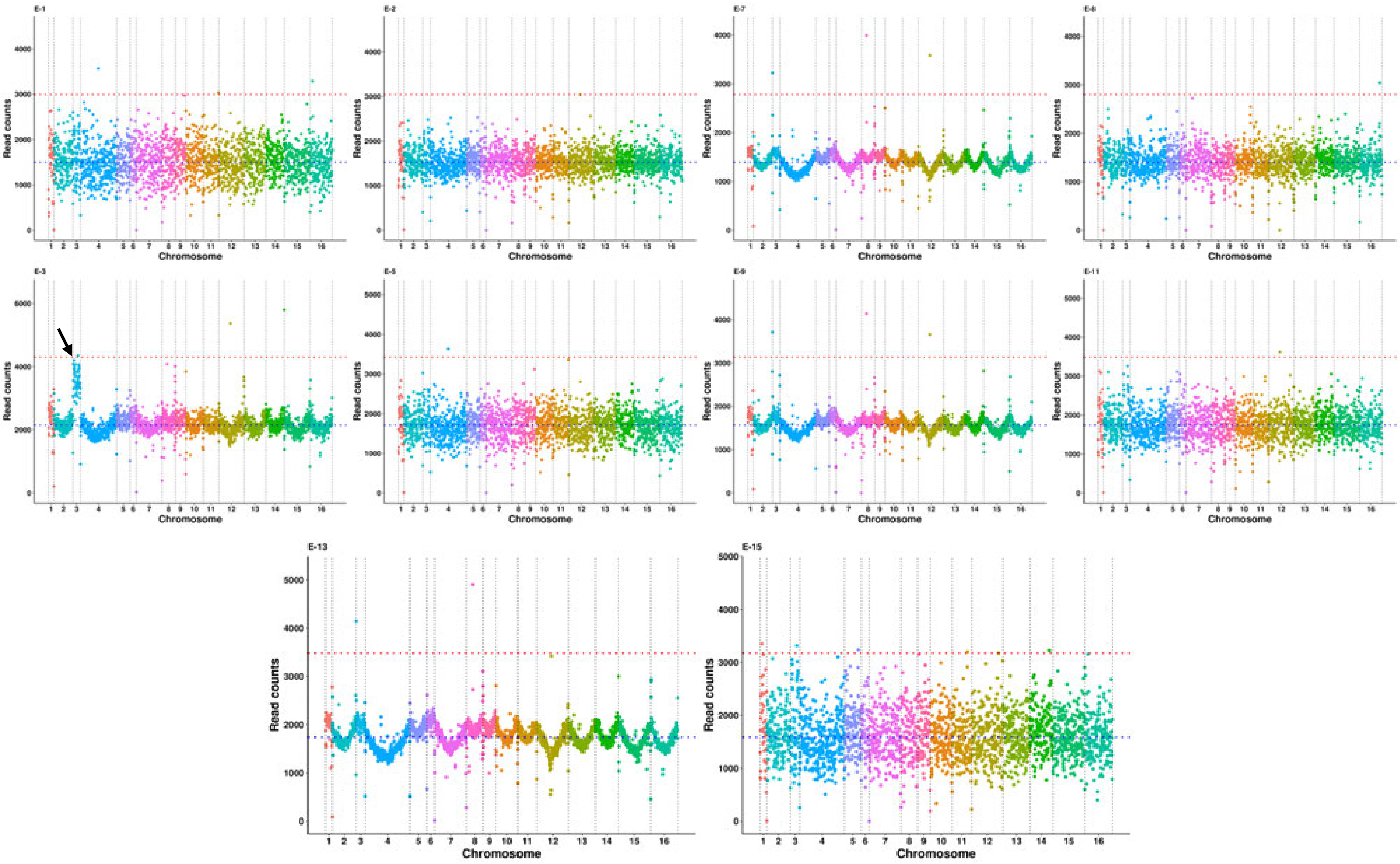

C

Ethanol – Aneuploidy profile

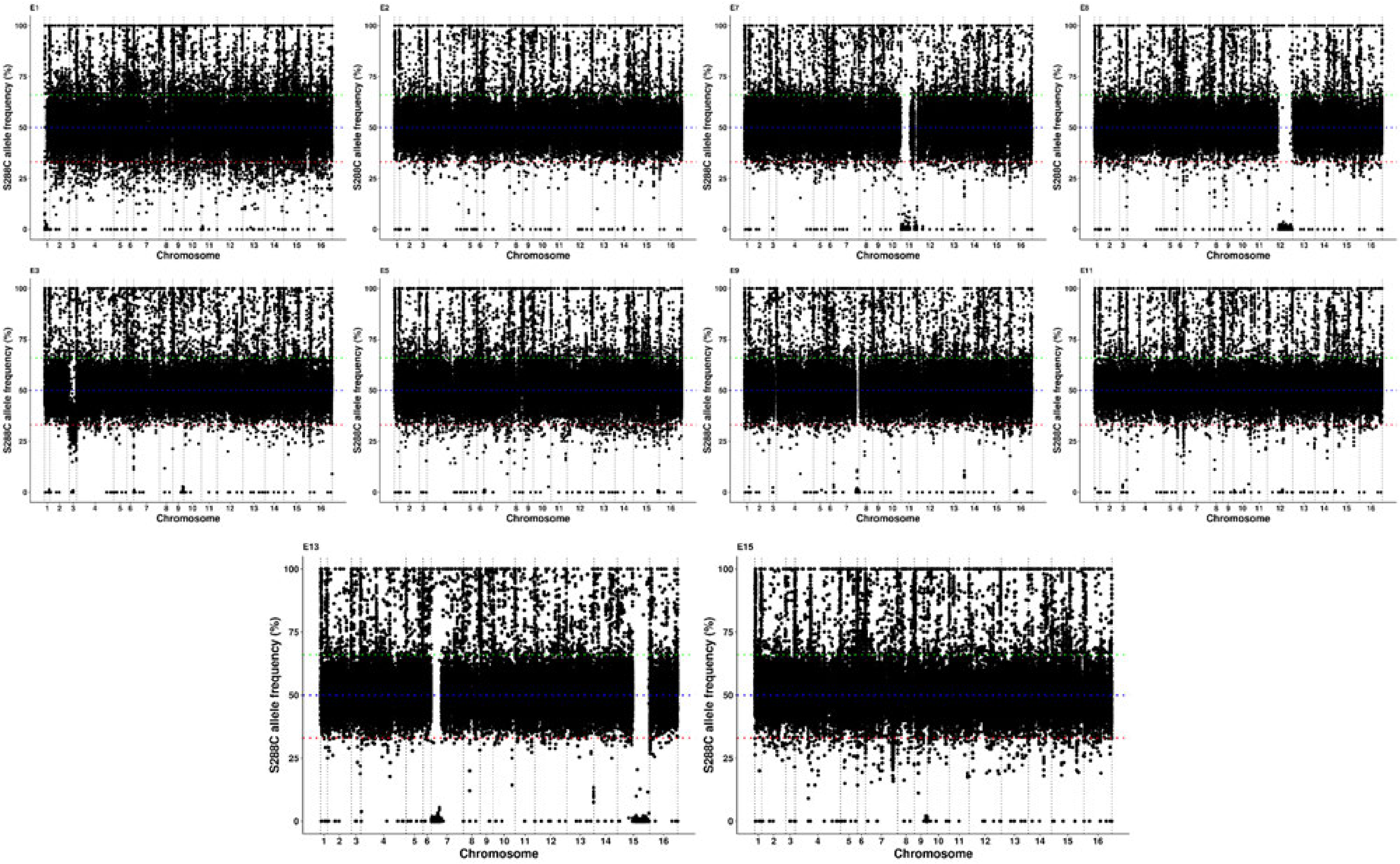

D

NaCl– Aneuploidy profile

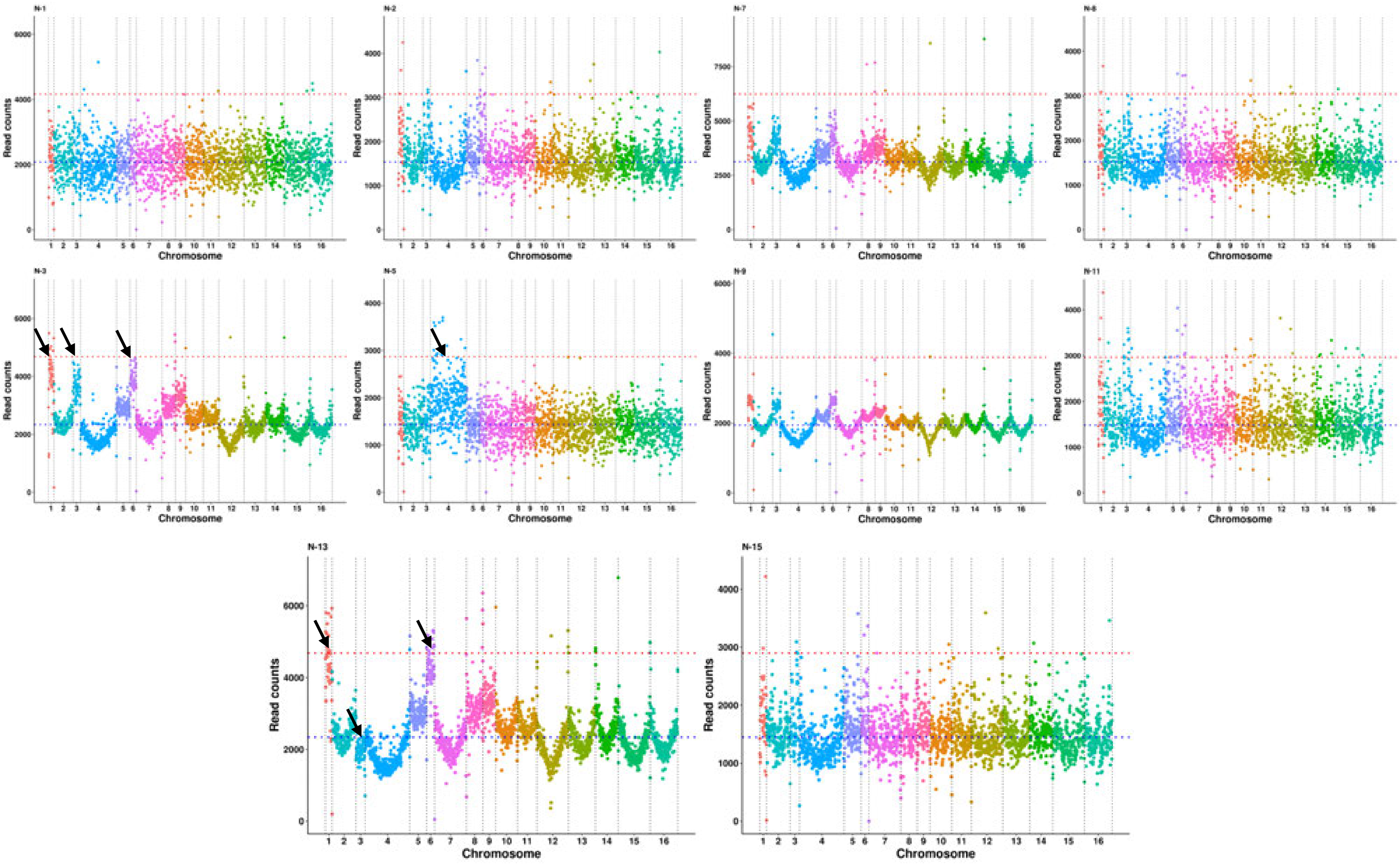

D

NaCl– Aneuploidy profile

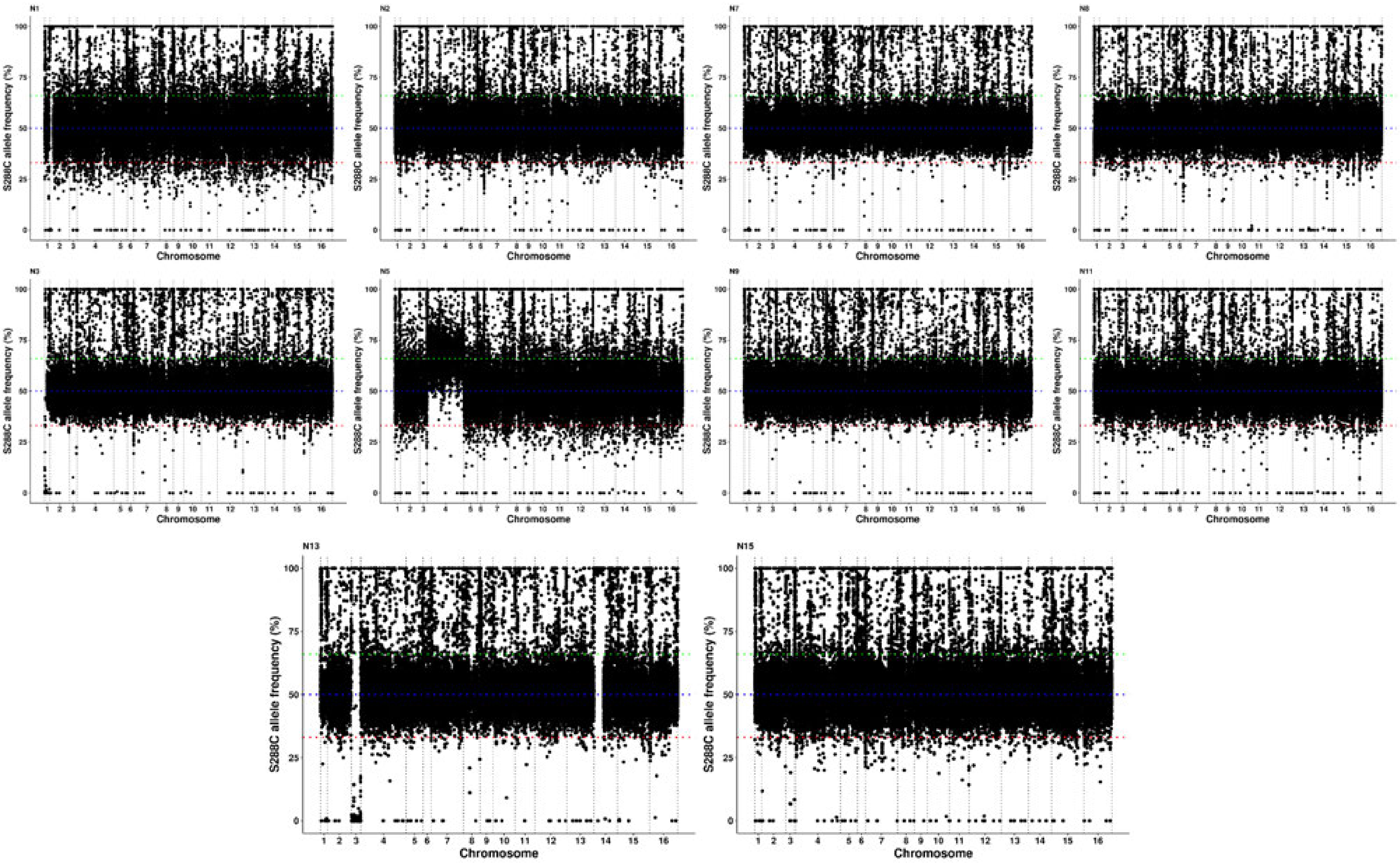

E

<u>High temperature – Aneuploidy profile</u>

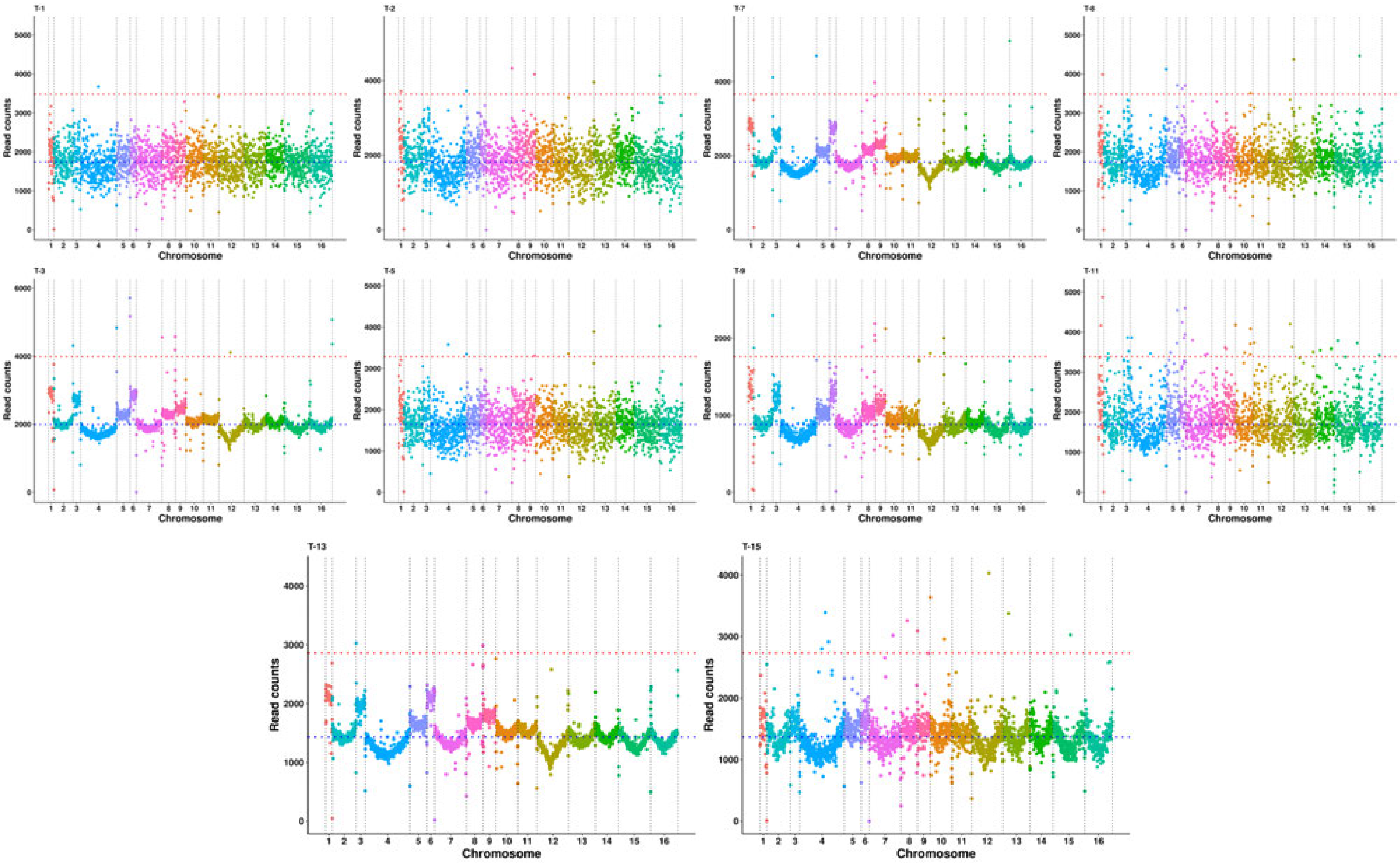

E

High temperature – Aneuploidy profile

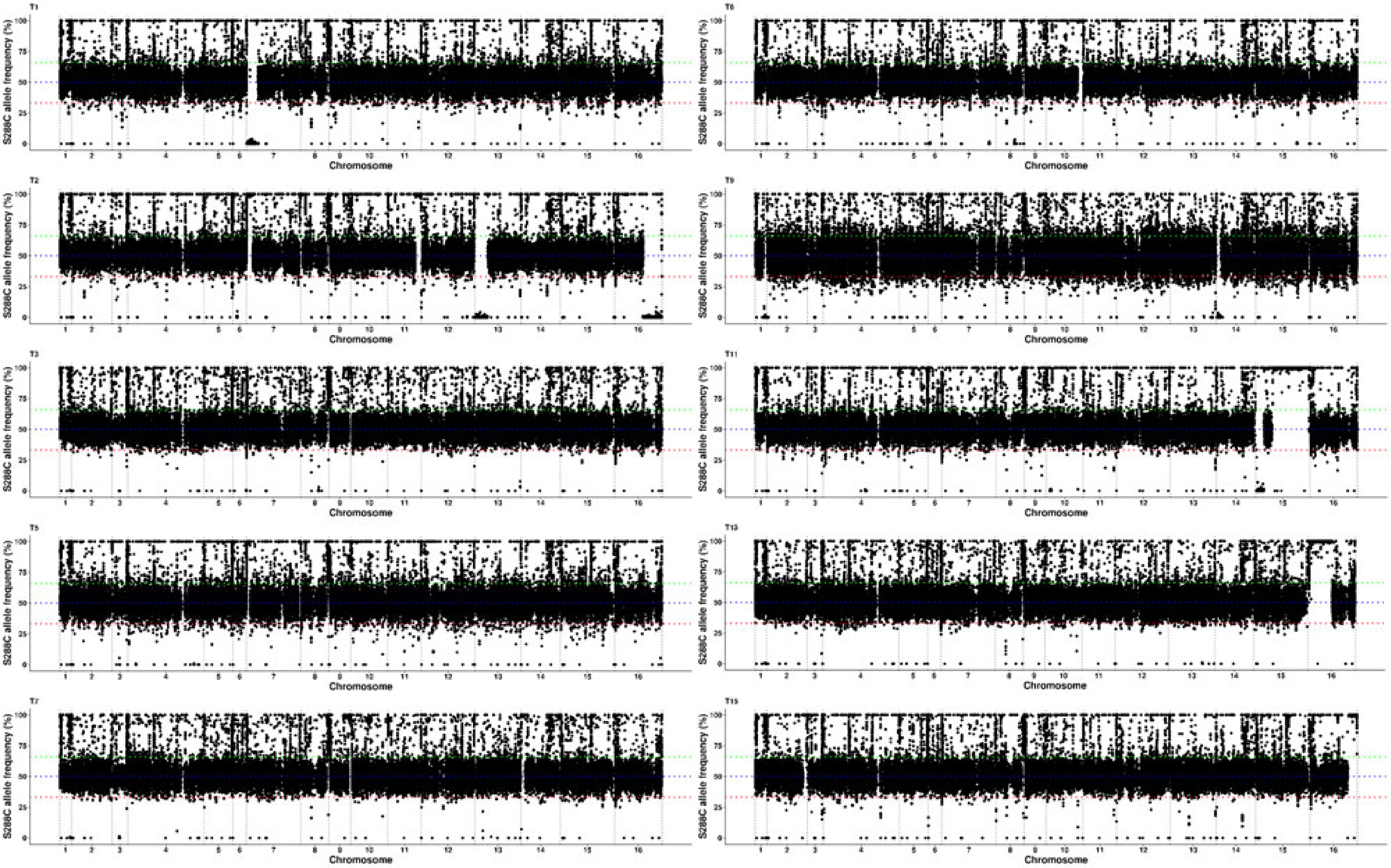

F

CR – Aneuploidy profile

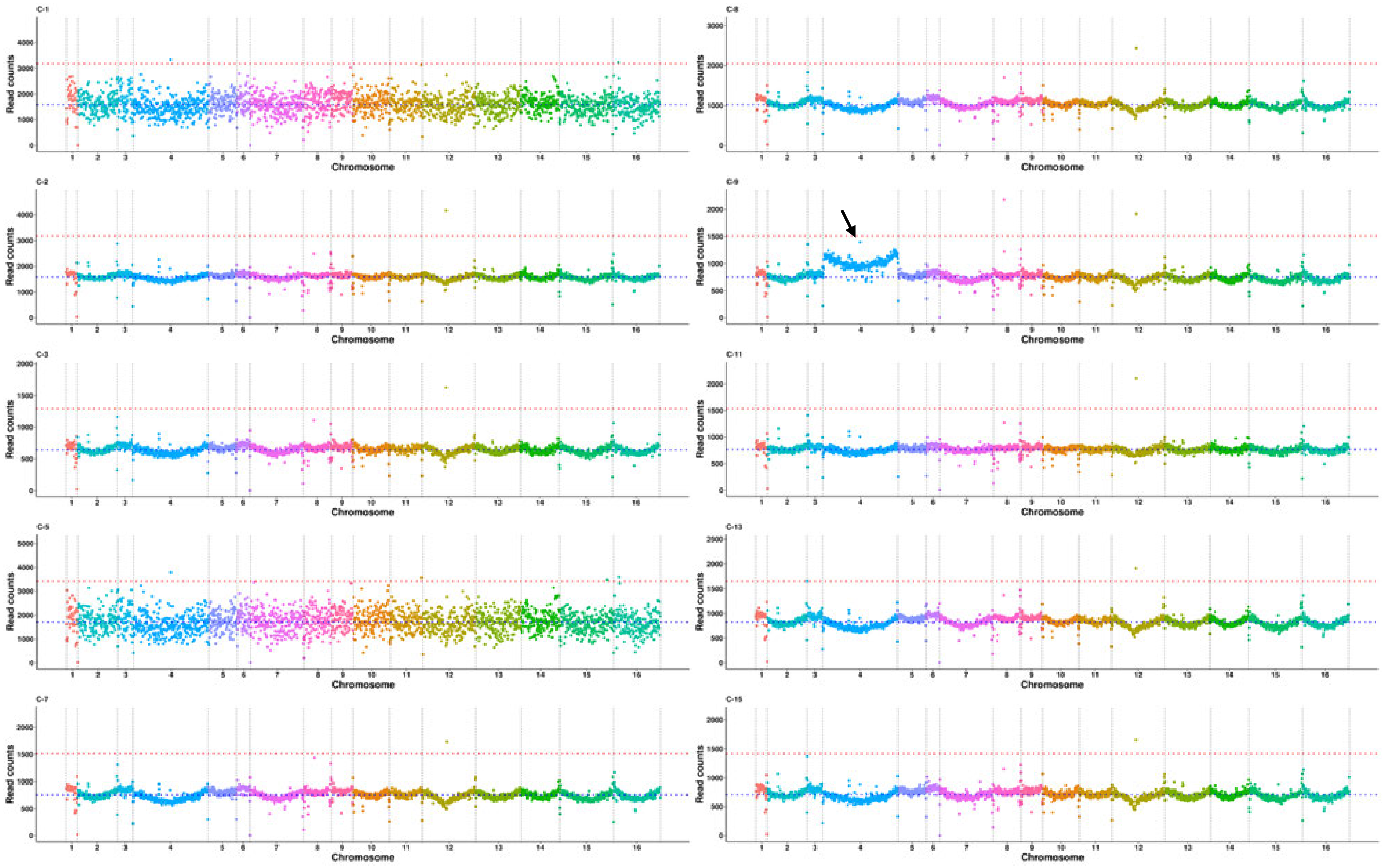

F

CR – Aneuploidy profile

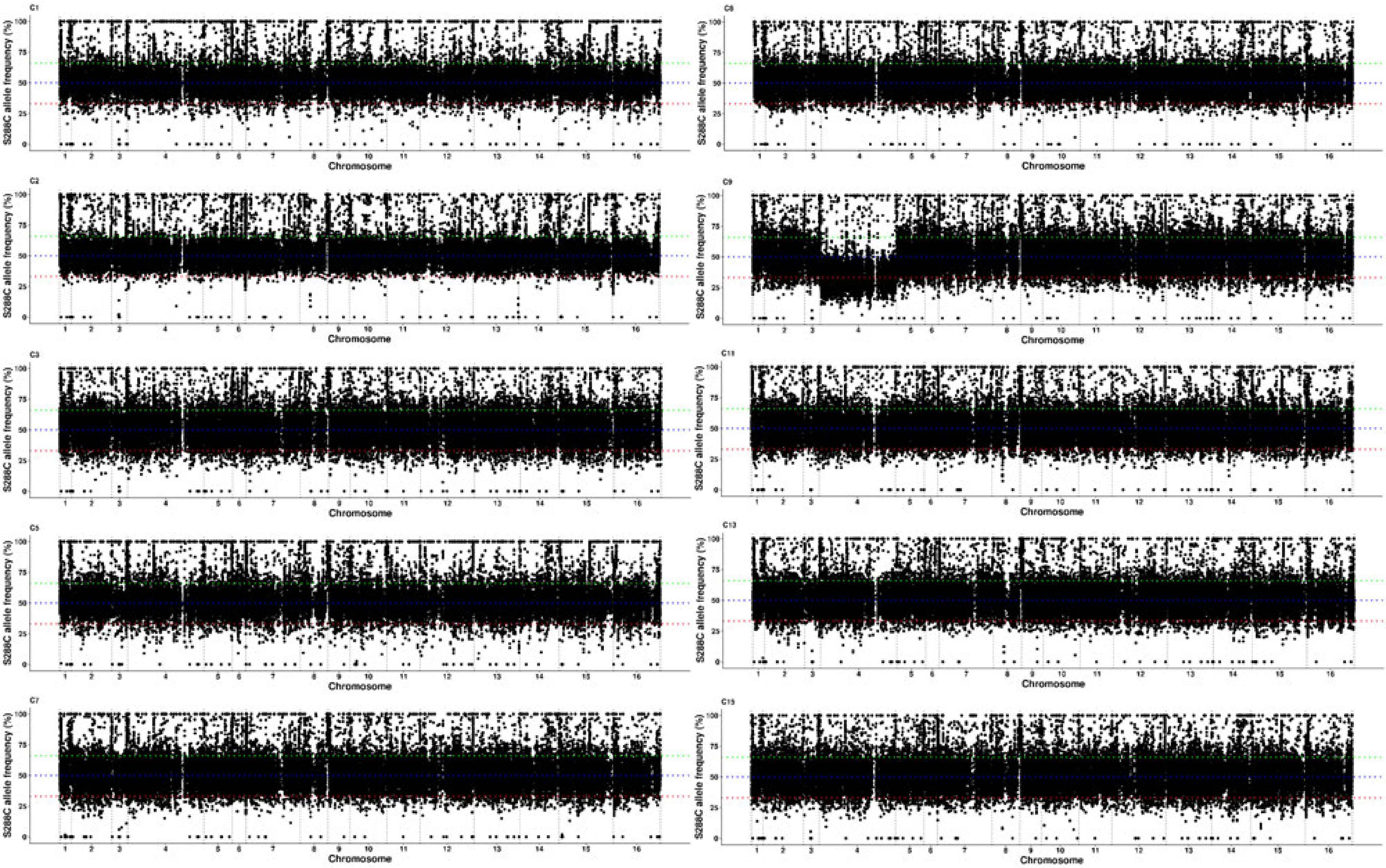

G

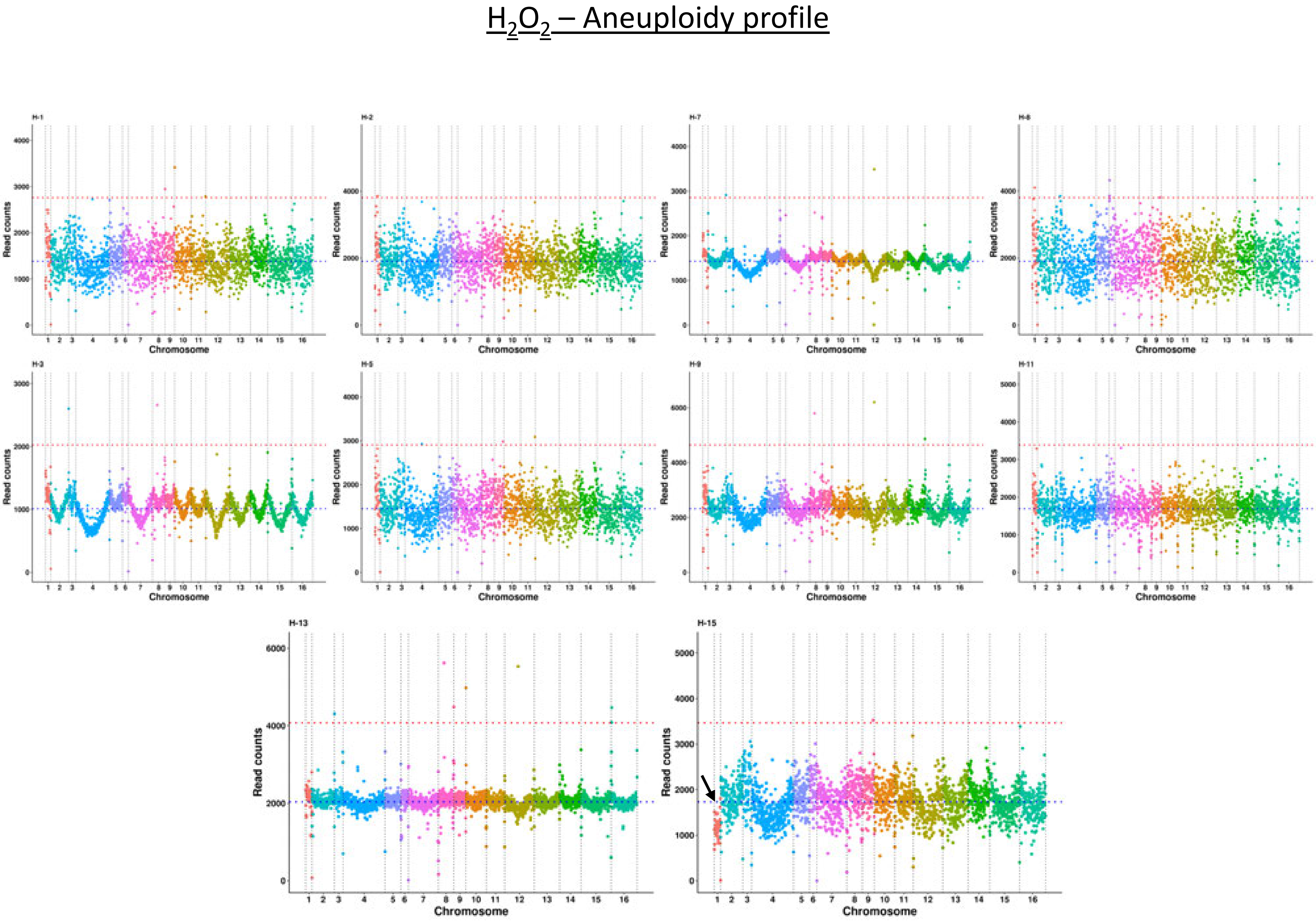

H_2O_2 – Aneuploidy profile
H-1
H-2
H-7
H-8
H-3
H-5
H-9
H-11
H-13
H-15
Read counts
Chromosome

G

H_2O_2 – Aneuploidy profile

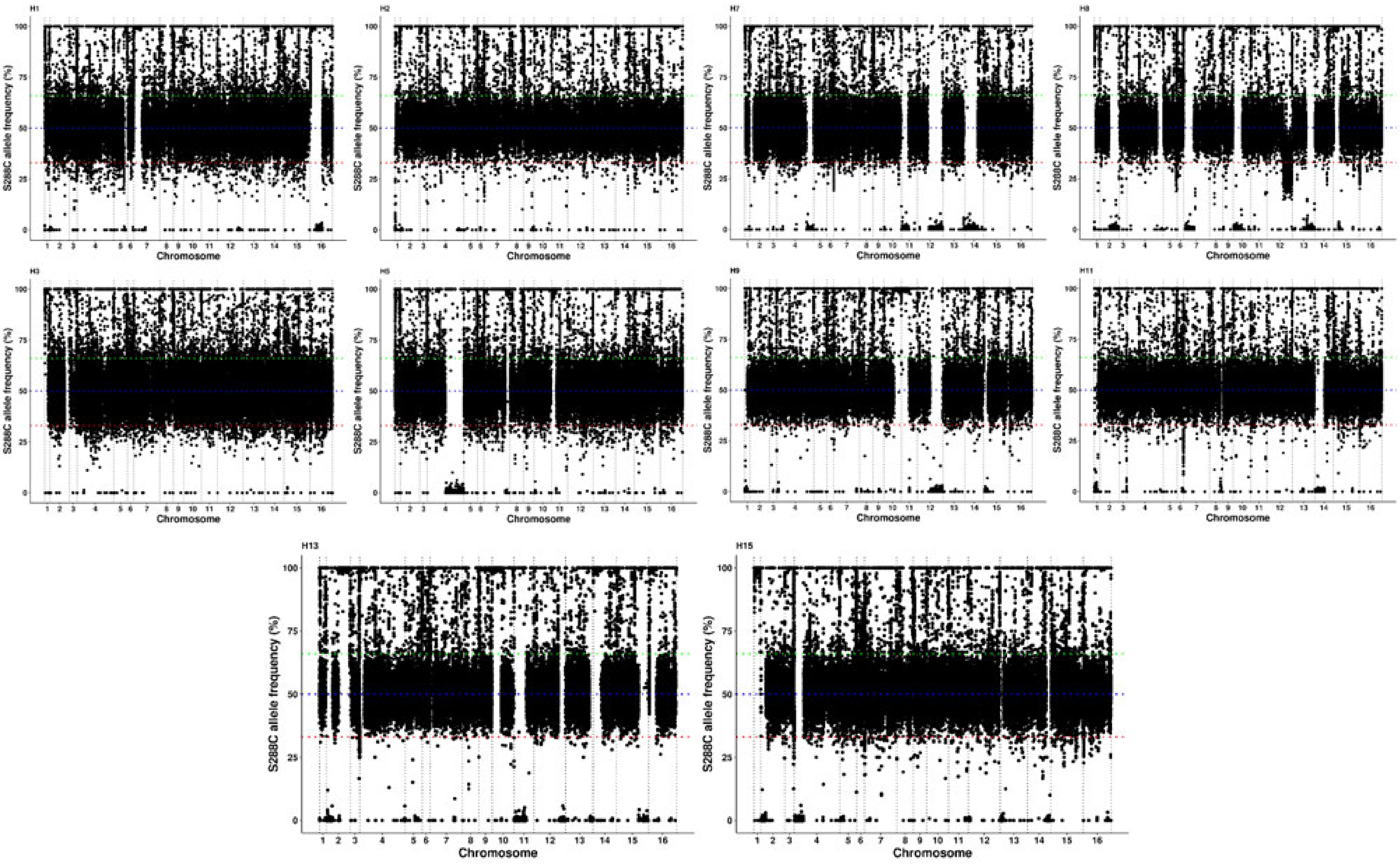

H

Blue light – Aneuploidy profile

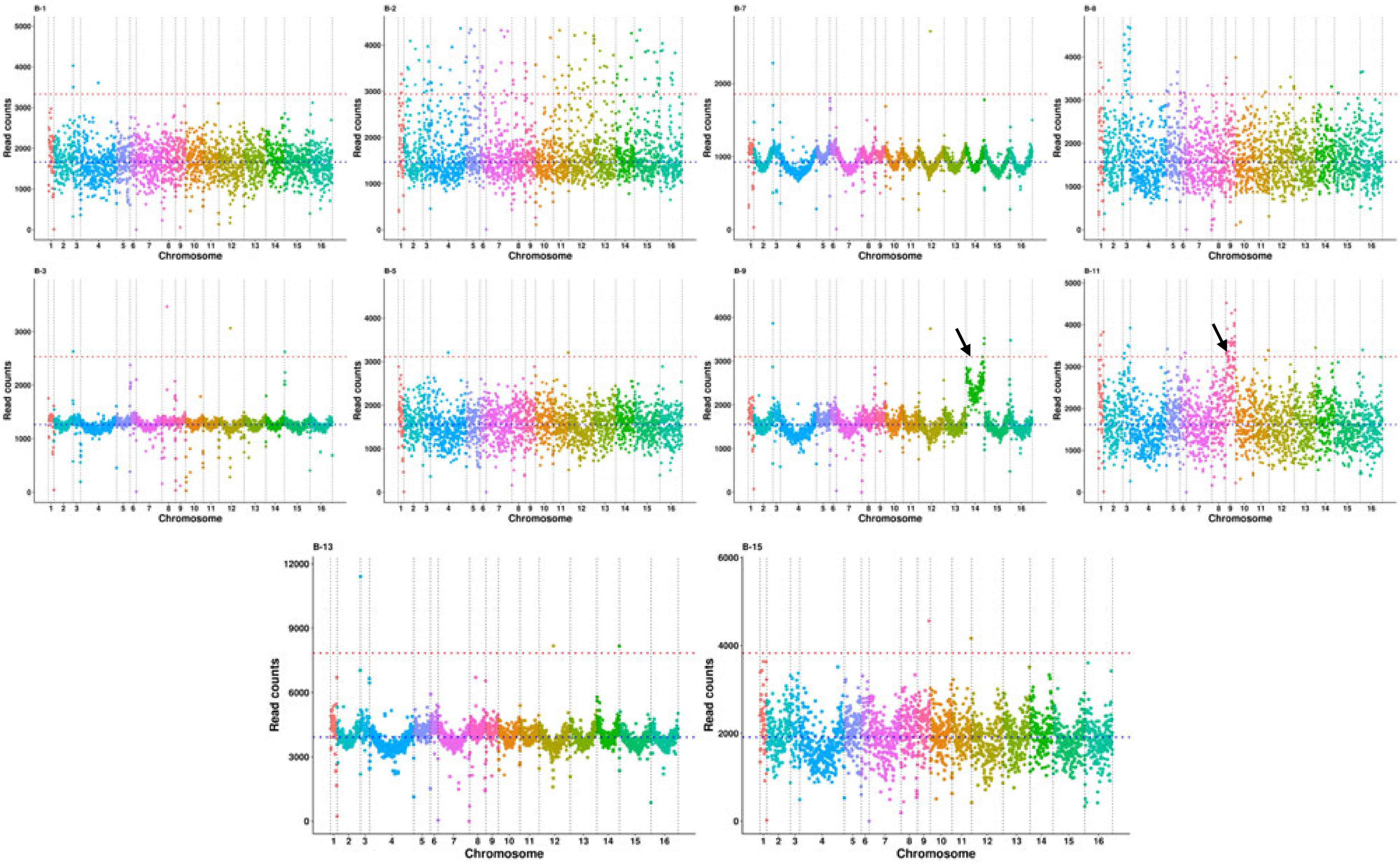

H

Blue light – Aneuploidy profile

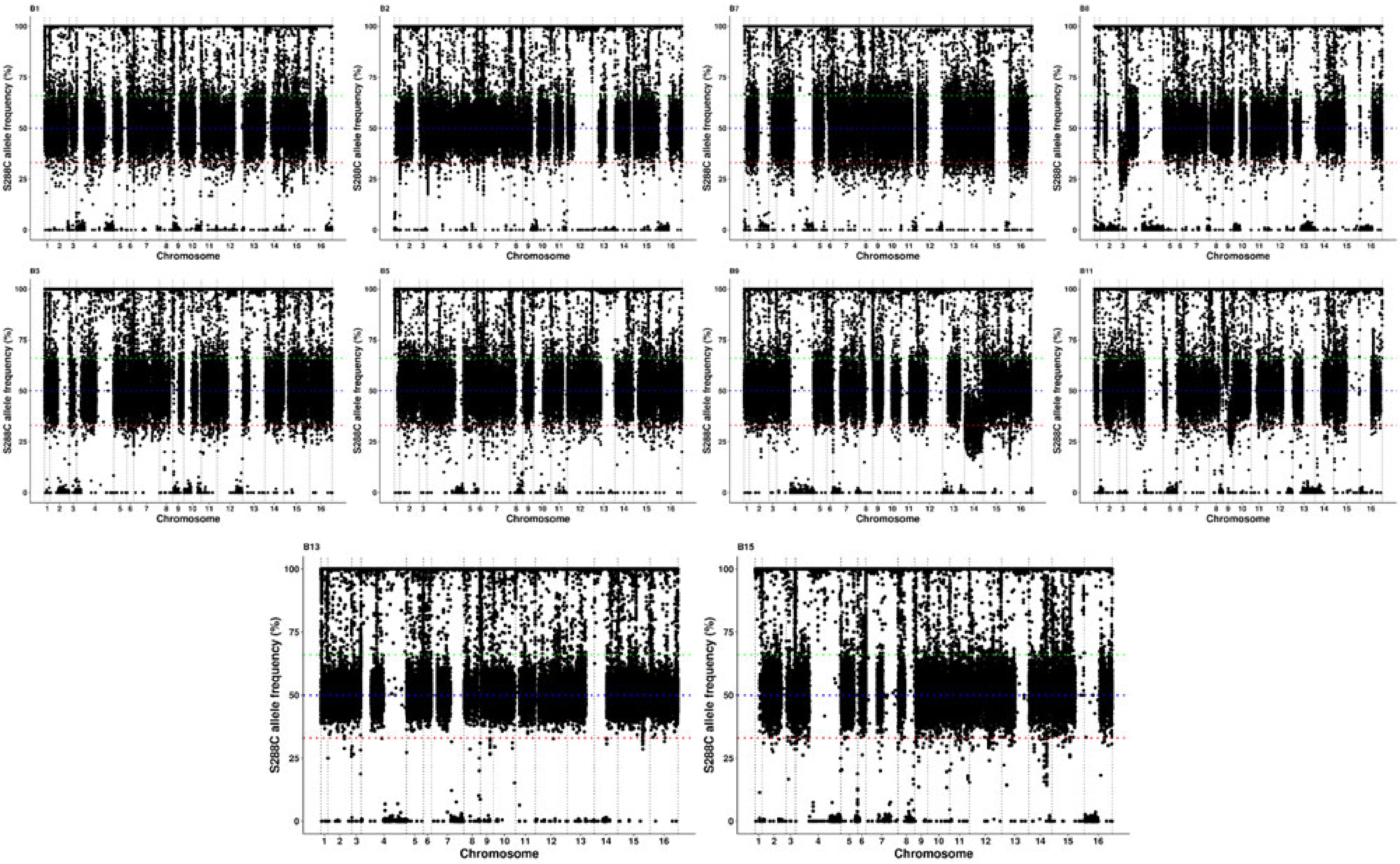

Supplement: S8 Fig — Read coverage and allele frequency plots are shown for B) YPD, C) Ethanol, D) NaCl, E) High temperature, F) calorie restriction, G) H2O2, H) Blue light. For the read coverage plots, colored dots show read counts in 5 kb bin sizes. The median and 2x median read counts are represented by the horizontal, blue and red dotted lines respectively. For the allele frequency plots, black dots show the frequency for the S288c alleles (percentage). Blue, red and green dotted lines show 50% (2n), 33% (1n), 66%(3n) S288c allele frequency. Vertical lines show the chromosomal boundaries. Black arrows in the coverage plots show the aneuploidy events. (PDF) [file pgen.1011692.s008.pdf]
