# Supplementary figures and images for: Loss of Heterozygosity associated with ubiquitous environments in yeast
Source: PLoS Genet. 2025 May 12;21(5):e1011692. doi: 10.1371/journal.pgen.1011692 (PMC12068580; doi:10.1371/journal.pgen.1011692)

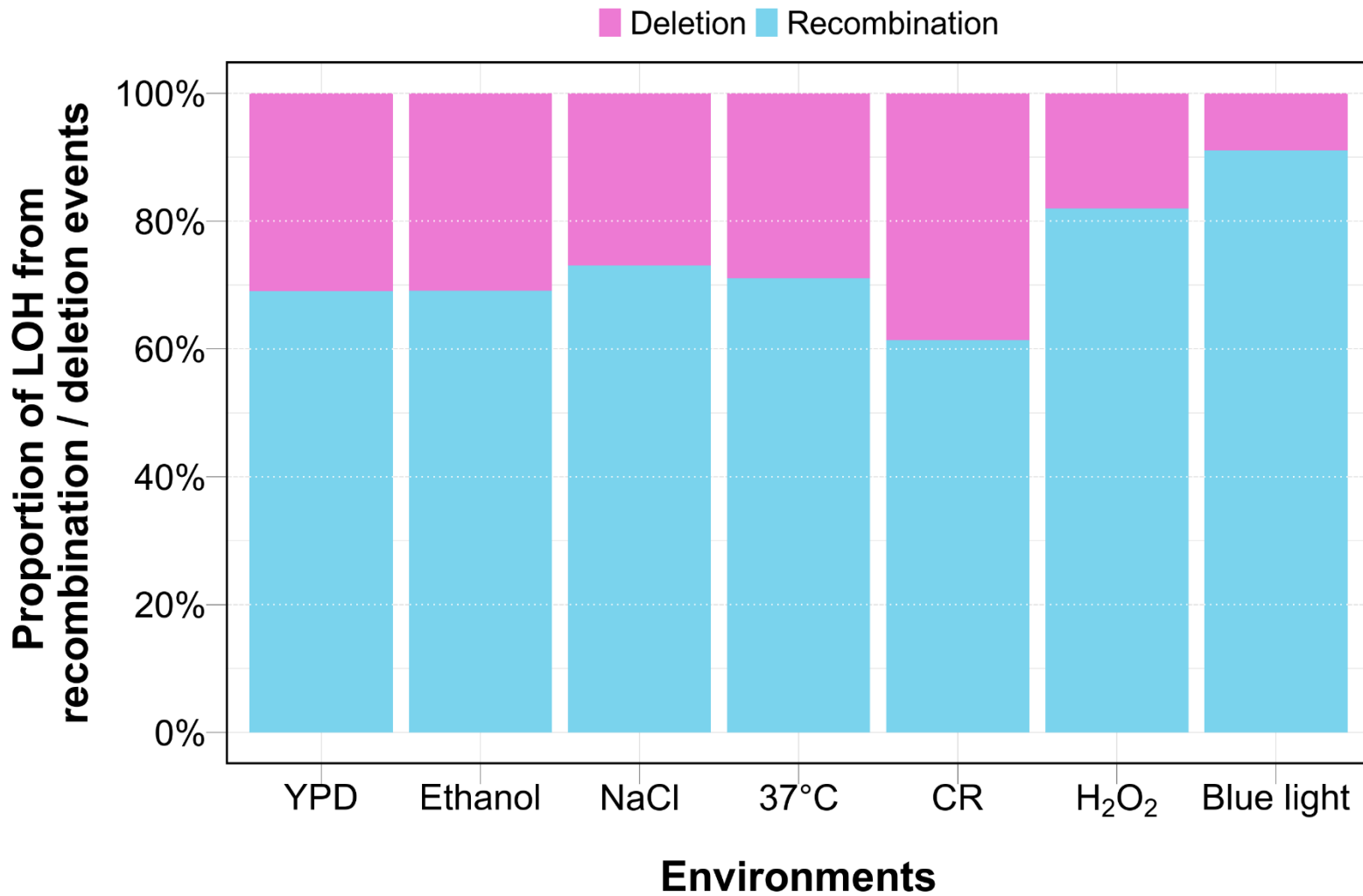

S9 Fig. Proportion of LOH events arising from recombination versus deletion events.

Supplement: S9 Fig — (PDF) [file pgen.1011692.s009.pdf]
