# Supplementary material for: Loss of Heterozygosity associated with ubiquitous environments in yeast
Source: PLoS Genet. 2025 May 12;21(5):e1011692. doi: 10.1371/journal.pgen.1011692 (PMC12068580; doi:10.1371/journal.pgen.1011692)
Supplement: S1 Table — For each condition, the number of bottlenecks was adjusted based on the initial growth rate to achieve ~1000 generations. Growth rates were estimated prior to starting the MA lines (initial growth rate) and at the end of the MA line propagation (final growth rate). The total number of generations propagated under a specific environment was estimated using the following formula: Total no. of generations = Total no. of bottlenecks × (Initial Generations per Bottleneck+Final Generations per bottleneck)/2. Here, the “Initial Generations per Bottleneck” represents the number of generations per bottleneck prior to MA line propagation, while the “Final Generations per Bottleneck” represents the corresponding number at the final bottleneck. (PDF) [file pgen.1011692.s010.pdf]

S1 Table. Final set of environmental conditions used for propagating MA lines. For each condition, the number of bottlenecks was adjusted based on the initial growth rate to achieve ~1000 generations.

Environment	Media	Growth rate (per hr)		Bottleneck interval	Final number of bottlenecks	Total generations
		Initial	Final			
**YPD**	1% yeast extract, 2% peptone and 2% glucose @ 30^0 C	0.543	0.562	24 h	53	1013.84
**Ethanol**	YPD with 6% ethanol @ 30^0 C	0.305	0.294	48 h	49	1015.95
**NaCl**	YPD with 500 mM NaCl @ 30^0 C	0.367	0.348	36 h	55	1021.34
**High temperature (37^0C)**	1% yeast extract, 2% peptone and 2% glucose @ 37^0 C	0.568	0.471	36 h	37	998.45
**CR**	1% yeast extract, 2% peptone and 0.05% glucose @ 30^0 C	0.275	0.261	48 h	52	965.3
**H_2O_2**	YPD with 3 mM H_2O_2 @ 30^0 C	0.501	0.493	36 h	42	1083.95
**Blue light**	1% yeast extract, 2% peptone and 2% glucose @ 30^0 C, 470 nm blue light at 500 µmol·m−2·s−1	0.497	0.347	36 h	43	942.16

Growth rates were estimated prior to starting the MA lines (initial growth rate) and at the end of the MA line propagation (final growth rate). The total number of generations propagated under a specific environment was estimated using the following formula: $\text{Total no. of generations} = \text{Total no. of bottlenecks} \times (\text{Initial Generations per Bottleneck} + \text{Final Generations per bottleneck})/2$. Here, the "Initial Generations per Bottleneck" represents the number of generations per bottleneck prior to MA line propagation, while the "Final Generations per Bottleneck" represents the corresponding number at the final bottleneck.
