# Supplementary material for: Loss of Heterozygosity associated with ubiquitous environments in yeast
Source: PLoS Genet. 2025 May 12;21(5):e1011692. doi: 10.1371/journal.pgen.1011692 (PMC12068580; doi:10.1371/journal.pgen.1011692)
Supplement: S11 Table — (PDF) [file pgen.1011692.s020.pdf]

S11 Table. Primers for RT-qPCR to analyze the expression of candidate genes under blue light exposure.

Primer pairs	DNA sequences (5' to 3')
POL3 FP<br>*POL3* RP	TATCTTTACGTCCCAGCGCC<br>TTGCTCCTGATCGTTAGCGTC
REV3 FP<br>*REV3* RP	TAAAGACCGTGTGCAGGACG<br>TGCCTGCATCGGAAGTGTAAC
RAD7 FP<br>*RAD7* RP	AAAAGCTTCCGAACTTGAAATCA<br>CTCCCATGTGTCCTCGTTGA
RAD26 FP<br>*RAD26* RP	GGAACCCATCTACTGACATGCA<br>GATACCTCTCTTTTCTGCCCAATC
RAD30 FP<br>*RAD30* RP	GCCTTTTTTGCACAGGTTGAG<br>CGCAGACTACCGGATCTTCTTT
RAD51 FP<br>*RAD51* RP	CTCTTTTGGGTGGTGGTGTG<br>ACCAATCTTACCGGCCTGAA
YAP1 FP<br>*YAP1* RP	ACACACCAAACTCCTCCACT<br>TGGGTTTCTTGGGAATGGGA
PIF1 FP<br>*PIF1* RP	CACTGCTTCCACGGGTTTAG<br>CATCGACAACCAAAGCACCA
POL4 FP<br>*POL4* RP	TTGTTGCAAGTGGGATGAGC<br>TCTATGTTCGGGTTCAGCGT
MSH2 FP<br>*MSH2* RP	CAATTGGACCCTGTAACGGC<br>CAATGTTCCCTGGAGATGCG
GAPDH FP<br>*GAPDH* RP	TTTCCCACGATGACAAGCAC<br>TGGAGCAGTGATGACAACCT
